# Fbxo2 suppresses prostate cancer progression by regulating YTHDF2 ubiquitination and degradation

**DOI:** 10.1038/s41419-025-08396-0

**Published:** 2025-12-29

**Authors:** Xinyu Xu, Guangcheng Dai, Chun-Ling Liu, Qiu Yao, Xiaowei Cai, Yang Wang, Zeyu Chen, Kang Liu, Jin Zhu, Jia Ma, Zhiwei Wang, Boxin Xue, Lixia Wang

**Affiliations:** 1 Department of Urology, The Second Affiliated Hospital of Soochow University, Suzhou, Jiangsu China; 2 Sir Run Run Hospital, Nanjing Medical University, Nanjing, Jiangsu China; 3 Center for Reproductive Medicine, the Second Affiliated Hospital of Soochow University, Suzhou, Jiangsu China; 4 Department of Biochemistry and Molecular Biology, School of Laboratory Medicine, Bengbu Medical University, Bengbu, Anhui China; 5 State Key Laboratory of Radiation Medicine and Protection, Soochow University, Suzhou, China

**Keywords:** Prostate cancer, Ubiquitylation

## Abstract

Deregulation of E3 ubiquitin ligases is associated with increased proliferation and metastasis in prostate cancer (PCa); however, the underlying mechanisms remain largely unclear. This study aimed to explore the role of Fbxo2, a SKP1-Cullin-F-box (SCF) E3 ubiquitin ligase, in PCa progression. Analysis of prostate tissue samples revealed that Fbxo2 is downregulated in PCa, and higher Fbxo2 expression correlates with better patient prognosis. Functional assays conducted both in vitro and in vivo demonstrated that Fbxo2 reduces cell proliferation and metastasis in PCa. Using co-immunoprecipitation mass spectrometry (co-IP-MS), co-IP, western blotting, and ubiquitin assays, we identified that m6A reader YTHDF2, an oncoprotein that is upregulated in PCa, was a substrate of Fbxo2-mediated degradation. Notably, Fbxo2 mutants lacking the C-terminal region were less effective in promoting YTHDF2 ubiquitination and destruction. Furthermore, lysine 286 (K286) of YTHDF2 was identified as the key ubiquitination site. A series of rescue experiments revealed that silencing or overexpressing YTHDF2 modulated the effects of Fbxo2 knockdown or overexpression, confirming their functional interplay. Mechanistically, YTHDF2 enhanced the PCa progression and metastasis by modulating the m6A methylation of *CDKN1C* mRNA. Together, these findings suggest that Fbxo2 axis may serve as a potential prognostic marker and therapeutic target in PCa.

## Introduction

Prostate cancer (PCa) is one of the most common malignancies in men and is recognized as the second leading cause of cancer-related mortality among the male population globally [1]. It is projected that ~299,010 new cases of PCa and 65,790 related deaths will occur in the United States in 2024, accounting for over 29% of all male cancer diagnoses [2]. Over the past decade, significant breakthroughs have been made in the clinical treatment of PCa, encompassing surgical resection, chemotherapy, immunotherapy, and targeted therapy [3]. However, the prognosis for patients with high-risk PCa and castration-resistant PCa (CRPC) remains poor [4]. Once metastasis occurs, the five-year survival rate for PCa drops sharply to ~30% [5, 6]. Therefore, there is an urgent need to further elucidate the molecular and cellular mechanisms underlying prostate cancer progression.

Ubiquitination represents a post-translational modification mediated by a cascade of three enzymes: E1 (activating enzyme), E2 (conjugating enzyme), and E3 (ligase enzyme) [7]. Recent studies suggest that ubiquitination plays a critical role in regulating a number of biological processes, including transcription, cell cycle progression, and various signaling pathways [8]. The largest family of E3 ubiquitin ligases is the Ring-finger protein family, among which the SCF (Skp1-Cullin-F-box)-type ubiquitin ligases are the most well-characterized [9]. F-box proteins, the substrate-recognition components of SCF complexes, regulate numerous processes associated with tumor proliferation, metastasis, and invasion [10]. They exert their effects through the regulation of DNA damage responses, cell cycle control, epithelial-mesenchymal transition (EMT), and varieties of signaling pathways including AKT/PI3K, p53, BMP, NRF2, NF-κB and Hippo pathways [11–13]. Fbxo2, also called FBG1 or Fbs1, is a member of FBXO protein subfamily and specifically recognizes asparagine-linked high-mannose-type carbohydrate chains [14]. Recent studies have demonstrated that Fbxo2 promotes osteosarcoma progression through enhancing STAT3 phosphorylation by degradation of IL-6R [15]. In ovarian cancer, Fbxo2 targets the glycosylated SUN2 protein, thereby preventing apoptosis, promoting cell proliferation, and facilitating tumor progression [16]. Remarkably, one study found that Fbxo2 expression was elevated in tumor-infiltrating areas and was associated with synaptic signaling pathways [17]. However, the function of Fbxo2 in prostate cancer remains poorly understood and warrants further investigation.

N6-methyladenosine (m6A) binding protein YTHDF2 selectively binds to the m6A consensus motif and facilitates the degradation of mRNA [18, 19]. Previous studies have shown that YTHDF2 is elevated in various tumor types, where it promotes cancer progression and metastasis, thereby accelerating the occurrence of multiple malignancies [20–22]. Conversely, in hepatocellular carcinoma, YTHDF2 acts a tumor suppressor by targeting EGFR, SERPINE2, and IL11 at mRNA levels [23, 24]. Ubiquitination has been identified as a crucial regulatory mechanism influencing YTHDF2 protein levels. It was claimed that SKP2 and FBW7 function as E3 ligases for YTHDF2, whereas OTUB1 acts as a deubiquitinase that blocks ubiquitin transfer to YTHDF2 [25–27]. To investigate the role of Fbxo2 in prostate cancer, we conducted a comprehensive series of experimental and bioinformatics analyses. Mass spectrometry and co-immunoprecipitation (co-IP) experiments were carried out, and identified YTHDF2 as a novel substrate of Fbxo2. Moreover, our results suggest that the Fbxo2-YTHDF2-*CDKN1C* axis may serve as a promising prognostic marker and therapeutic target in PCa.

## Results

### Fbxo2 is downregulated in PCa tissues and correlates with favorable prognosis

**Fig. 1:**
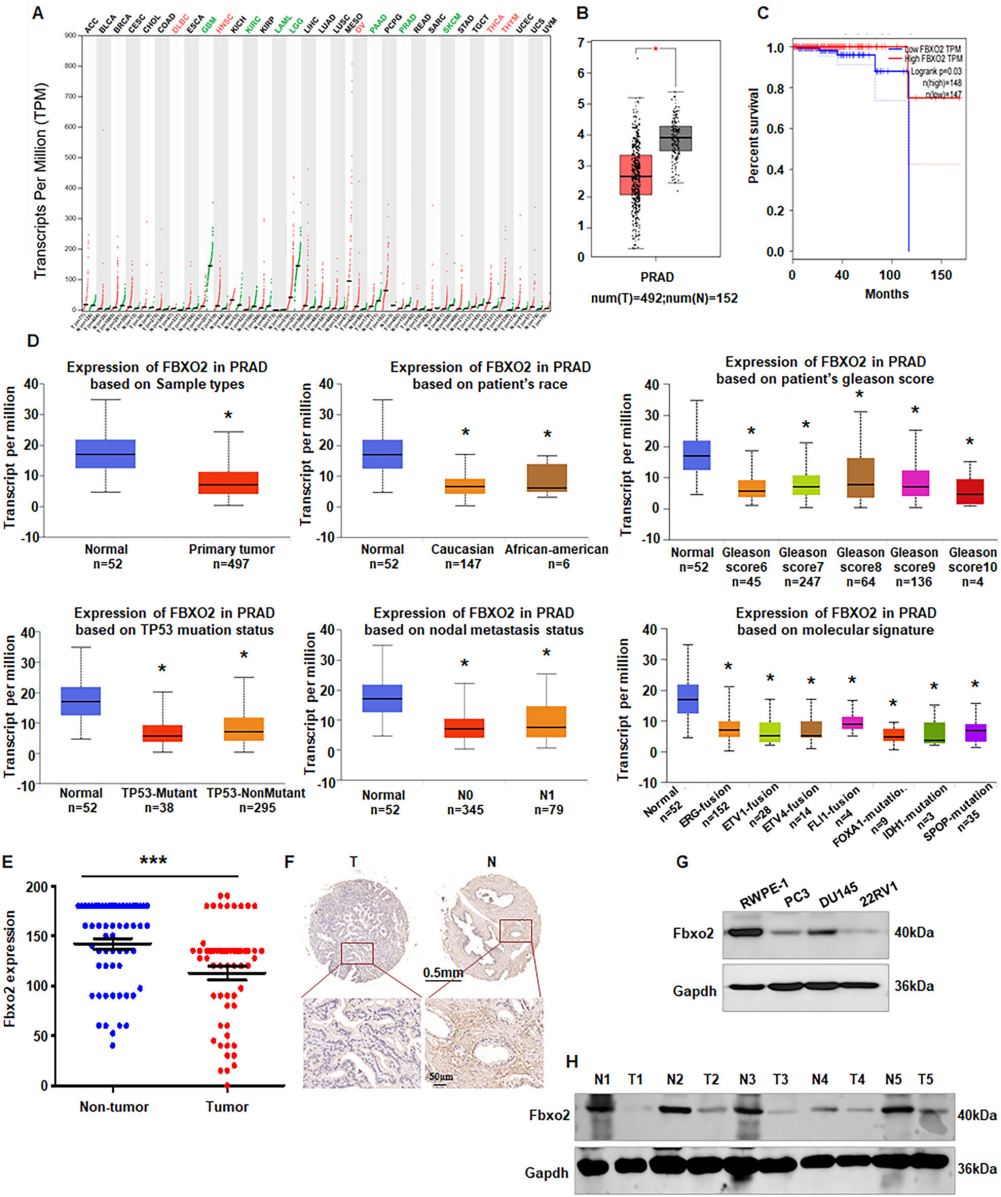
Upregulation of Fbxo2 correlates with favorable prognosis in PCa. **A**, **B** Fbxo2 expression in tumor tissues versus normal tissues across various cancer types, with a significant decrease observed in PCa, based on the GEPIA database. **C** Kaplan–Meier survival analysis showing overall survival (OS) of PCa patients stratified by Fbxo2 expression levels. **D** Correlation between Fbxo2 expression and clinicopathological features, including patient age, Gleason score, TP53 mutation status, nodal metastasis status, and molecular subtype, as indicated by the UALCAN database. **E**, **F** Representative immunohistochemistry (IHC) images and quantification of Fbxo2 expression in 60 paired PCa and adjacent non-tumorous tissue samples. **G** Western blot analysis of Fbxo2 protein expression in the normal prostate epithelial cell line (RWPE-1) and PCa cell lines. **H** Immunoblot analysis of Fbxo2 protein levels in freshly collected PCa tissues and matched adjacent normal tissues. *p < 0.05, ***p < 0.001.

Given the limited data on the role of Fbxo2 in PCa, we first investigated its expression in malignant versus non-cancerous prostate tissues in order to ascertain its clinical relevance. Analysis of the GEPIA database revealed that Fbxo2 expression was decreased in PCa tissues compared with normal prostate tissues (Fig. 1A, B). Moreover, higher expression of Fbxo2 was associated with improved prognosis in PCa patients (Fig. 1C). Consistently, data from the UALCAN database demonstrated that Fbxo2 expression correlated with several clinical characteristics, including patient age, Gleason score, TP53 mutation status, nodal metastasis status, and molecular signature in PCa (Fig. 1D). To further validate these results, we performed immunohistochemical (IHC) analysis on PCa tissues. The IHC staining confirmed that Fbxo2 expression was lower in tumor tissues compared to adjacent normal tissues (Fig. 1E, F). Fbxo2 expression was notably decreased in three prostate cancer cell lines (Fig. 1G, Supplementary Fig. S1A). Immunoblotting of freshly collected clinical specimens demonstrated markedly reduced Fbxo2 protein levels in PCa tissues relative to paired normal tissues (Fig. 1H).

### Fbxo2 inhibits cell proliferation and motility and induces apoptosis

**Fig. 2:**
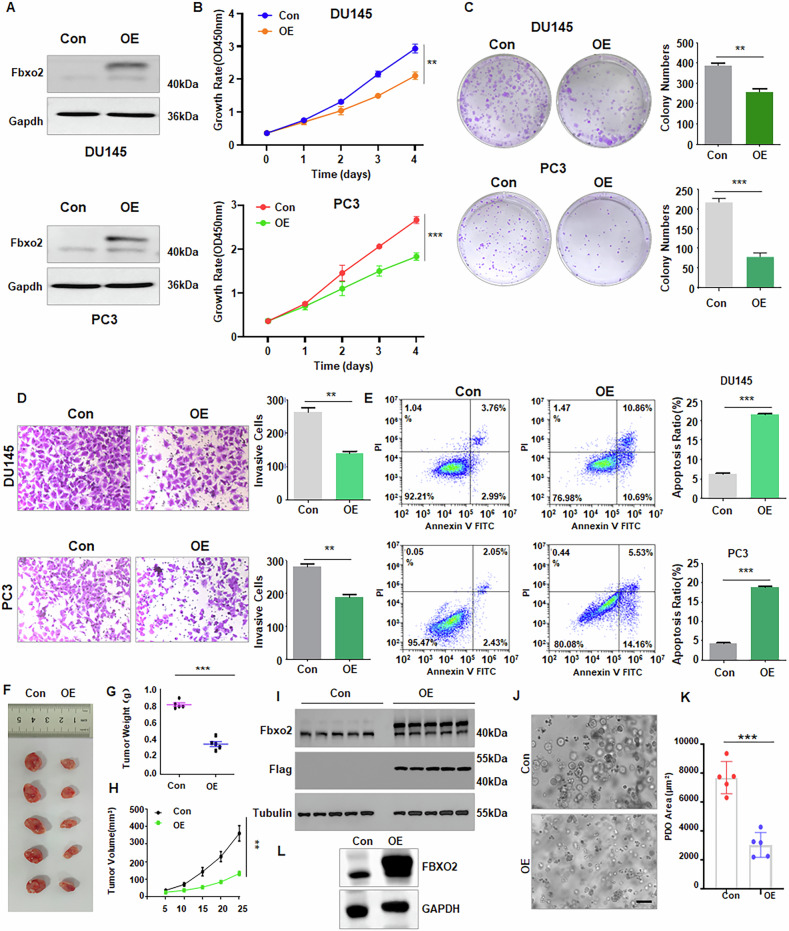
Overexpression of Fbxo2 inhibits cell proliferation and invasion in PCa. **A** Western blot was performed to detect Fbxo2 expression in PC3 and DU145 cells transfected with Flag-Fbxo2 or empty vector lentivirus. **B**, **C** Cell viability and proliferation were assessed by CCK-8 and colony formation assays in Fbxo2-overexpressing PC3 and DU145 cells. **D** The impact of Fbxo2 overexpression on cell invasion was examined using the Transwell assay in PCa cells. **E** Apoptosis of Fbxo2-overexpressing PC3 and DU145 cells was measured by flow cytometric analysis. **F** Representative images of xenograft tumors formed by subcutaneous injection of Fbxo2-overexpressing DU145 cells in nude mice. **G**, **H** Tumor growth curves and tumor weights over a 25-day period. **I** Western blot analysis of Fbxo2 protein levels in excised xenograft tumors. **J** Representative images of PDOs to assess the effect of FBXO2 overexpression on tumor burden. Scale bars = 200 µm. **K** Quantification of the relative area of PDOs. **L** Western blotting of PDOs showing the transfection efficiency of FBXO2. *p < 0.05, **p < 0.01, ***p < 0.001.

To investigate the biological functions of Fbxo2 in PCa, we established stable Fbxo2-overexpressing PC3 and DU145 cell lines by transfecting cells with Flag-Fbxo2 lentivirus (Fig. 2A, Supplementary Fig. S1B). CCK-8 and colony formation assays revealed that overexpression of Fbxo2 significantly suppressed the proliferation of PCa cells (Fig. 2B, C). Transwell and wound-healing assays verified that Fbxo2-overexpresing PC3 and DU145 cells exhibited markedly reduced invasion and migration abilities (Fig. 2D, Supplementary Fig. S1C, D). Furthermore, flow cytometric analysis revealed that Fbxo2 overexpression induced higher levels of apoptosis in PCa cells (Fig. 2E). To evaluate the role of Fbxo2 in vivo, we developed a subcutaneous xenograft model. Consistent with our in vitro results, tumors formed by Fbxo2-overexpressing cells were significantly smaller in volume compared to those in the control group (Fig. 2F–I, Supplementary Fig. S1E). Notably, FBXO2 overexpression significantly suppressed the growth of prostate cancer patient-derived organoid (PDO) models (Fig. 2J–L). Taken together, these results suggest that upregulation of Fbxo2 significantly inhibits PCa cell proliferation and motility and induces cell apoptosis.

### Fbxo2 depletion promotes proliferation, motility and inhibits apoptosis

**Fig. 3:**
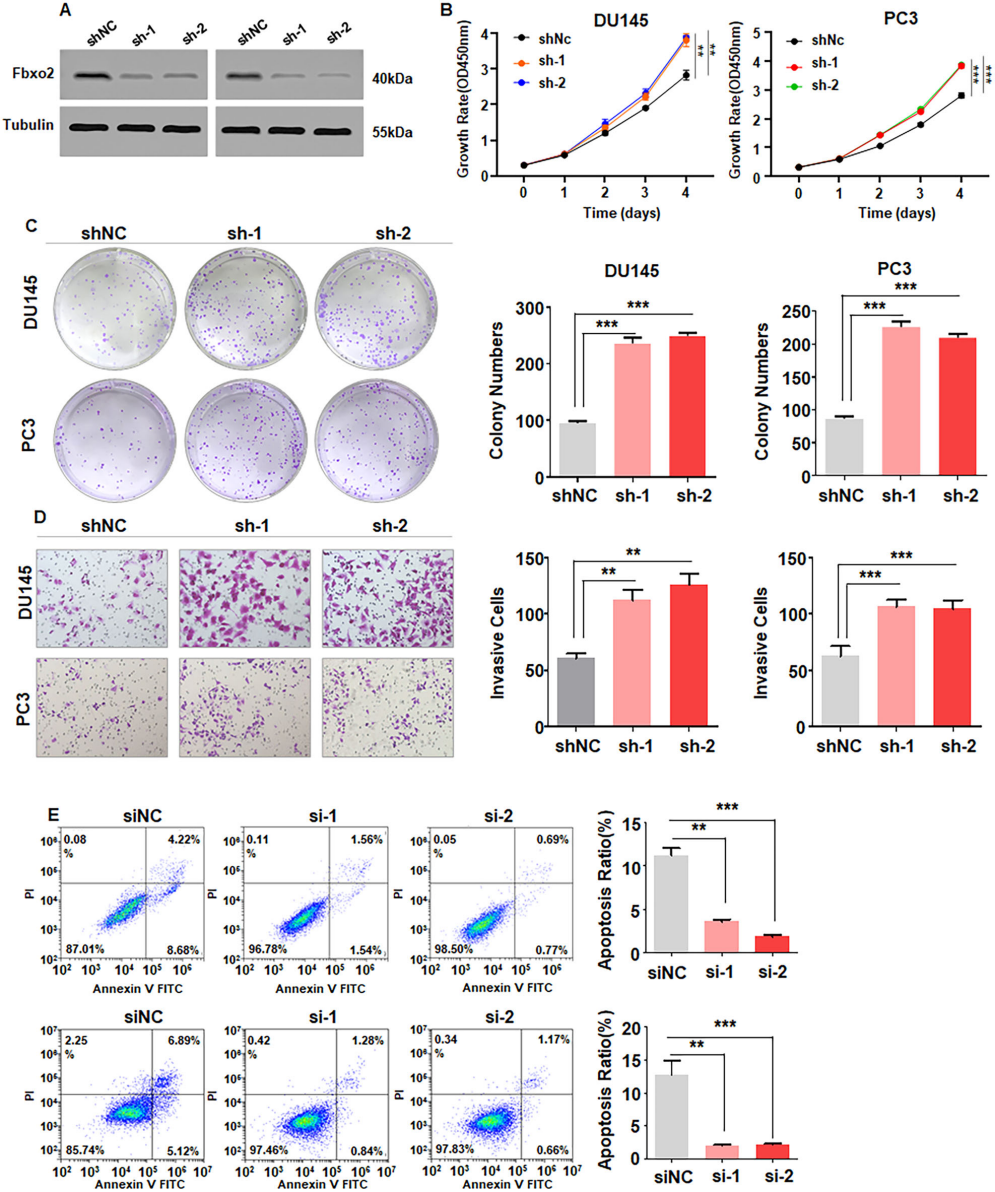
Silencing of Fbxo2 facilitates proliferation and motility in PCa cells. **A** Western blot analysis of Fbxo2 protein levels in PC3 and DU145 cells transfected with Fbxo2 shRNA or control shRNA lentivirus. **B**, **C** The viability of PC3 and DU145 cells infected with Fbxo2 shRNA lentivirus was assessed by using CCK-8 and colony formation assays. **D** The invasion capability of PC3 and DU145 cells treated with NC shRNA or Fbxo2 shRNA lentivirus was evaluated by Transwell assay. **E** Apoptotic rate was measured in PC3 and DU145 cells treated with Fbxo2 siRNA by flow cytometric analysis. *p < 0.05, **p < 0.01, ***p < 0.001.

To further investigate the functional role of Fbxo2, we constructed Fbxo2 knockdown models in PC3, DU145 and C4-2 cells using Fbxo2 shRNA lentiviral infection (Fig. 3A, Supplementary Fig. S1F). CCK-8 and colony formation assays verified that downregulation of Fbxo2 enhanced the proliferative capacity of PCa cells (Fig. 3B, C, Supplementary Fig. S1G). Transwell and wound-healing experiments revealed significantly increased cell invasive and migratory capabilities upon Fbxo2 silencing (Fig. 3D, Supplementary Figs. S1H, S2A–D). In addition, flow cytometric analysis showed that downregulation of Fbxo2 markedly reduced apoptosis in PCa cells (Fig. 3E). These findings collectively indicate that Fbxo2 functions as a tumor suppressor by inhibiting cell growth and motility in PCa.

### Fbxo2 targets YTHDF2 for ubiquitination and proteasomal degradation

**Fig. 4:**
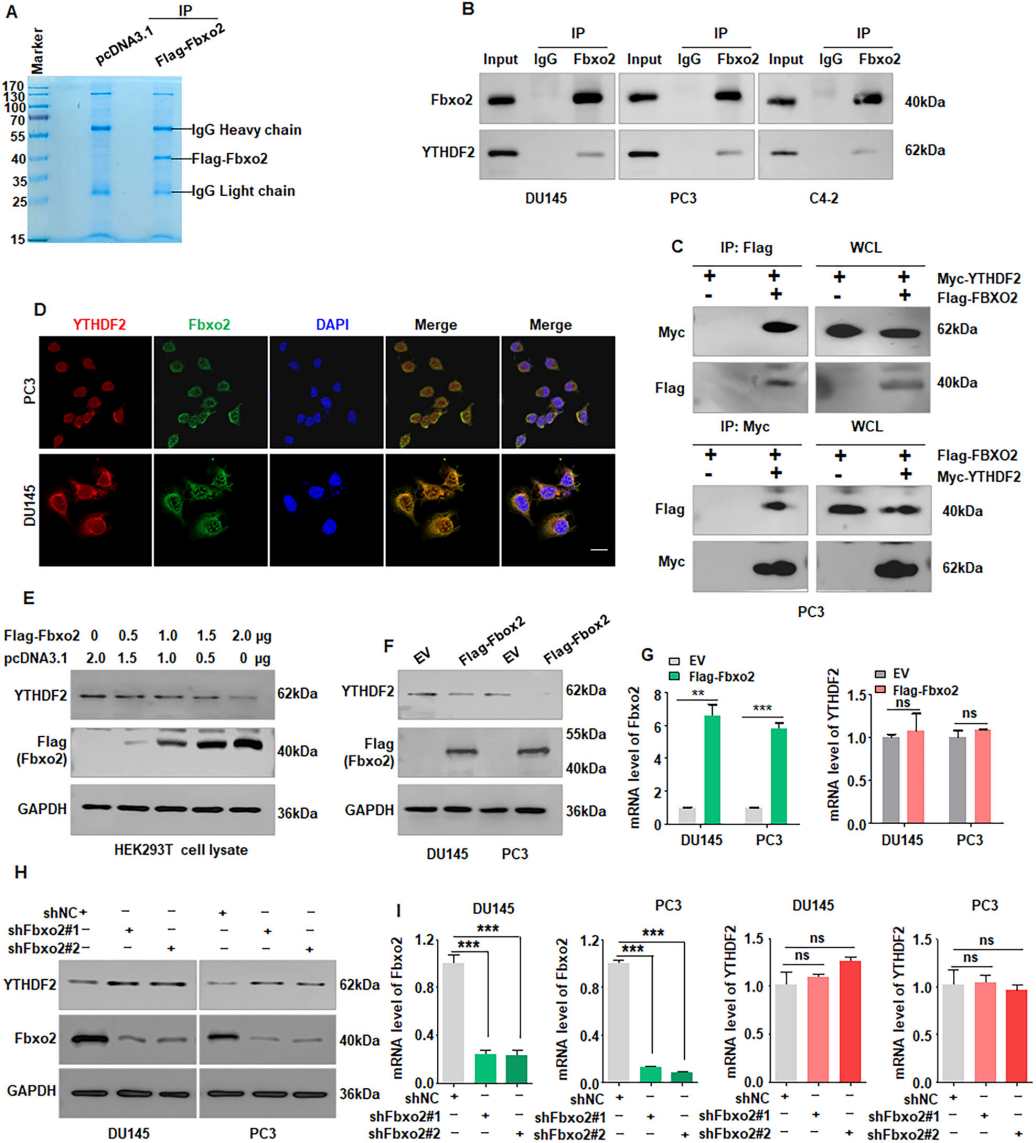
Fbxo2 binds to and degrades YTHDF2. **A** 293T cells were transfected with either Flag-Fbxo2 or empty vector (Vec) for 48 h and lysed in NP-40 buffer. Flag immunoprecipitates were analyzed by SDS-PAGE and Coomassie Brilliant Blue staining. **B** Co-immunoprecipitation (Co-IP) and Western blotting were used to detect the interaction between endogenous Fbxo2 and YTHDF2 in PC3, DU145 and C4-2 cells. **C** To confirm exogenous interactions, PC3 and DU145 cells were co-transfected with Flag-Fbxo2 and Myc-YTHDF2 plasmids for 48 h, followed by anti-Flag or anti-Myc immunoprecipitation and Western blotting. **D** The subcellular localization of Fbxo2 (green), YTHDF2 (red) and nuclei (DAPI, blue) in PC3 and DU145 cells were visualized using a confocal fluorescence microscopy. **E**, **F** Protein levels of YTHDF2 in 293T, PC3 and DU145 cells with or without Fbxo2 overexpression were assessed using Western blotting. **G** Using RT-PCR, the mRNA expression of YTHDF2 was assessed in PC3 and DU145 cells with or without Fbxo2 overexpression. **H**, **I** Western blot and RT-PCR analyses of YTHDF2 protein and mRNA levels, respectively, in PC3 and DU145 cells with or without Fbxo2 knockdown. *p < 0.05, **p < 0.01, ***p < 0.001.

To identify potential substrates of Fbxo2, we performed liquid chromatography-tandem mass spectrometry (LC-MS/MS) analysis. Fbxo2 immunocomplexes were isolated from DU145 cells transfected with Flag-Fbxo2 and subjected to LC-MS/MS (Fig. 4A, Supplementary Fig. S3A). Among the identified peptides, YTHDF2 was detected as a potential binding partner of Fbxo2 (Supplementary Fig. S3B). To validate this interaction, co-IP assays were performed and confirmed both endogenous and exogenous interactions between Fbxo2 and YTHDF2 in PC3, DU145 and C4-2 cells (Fig. 4B, C). Immunofluorescence staining further showed that Fbxo2 and YTHDF2 co-localized predominantly in the cytoplasm of PCa cells (Fig. 4D). Ectopic overexpression of Fbxo2 dramatically lowered YTHDF2 protein levels, while RT-qPCR results showed no influence on YTHDF2 mRNA levels (Fig. 4E–G). Consistently, we noticed that silencing of Fbxo2 led to increased YTHDF2 protein expression in PCa cells (Fig. 4H, Supplementary Fig. S3C–G) without affecting its mRNA levels (Fig. 4I). In addition, FBXO2 does not significantly affect IGF2BP3 protein levels (Supplementary Fig. S4A, B). These results indicate that Fbxo2 could affect the YTHDF2 protein levels via a post-transcriptional mechanism.

### Mechanism of YTHDF2 ubiquitination mediated by Fbxo2

**Fig. 5:**
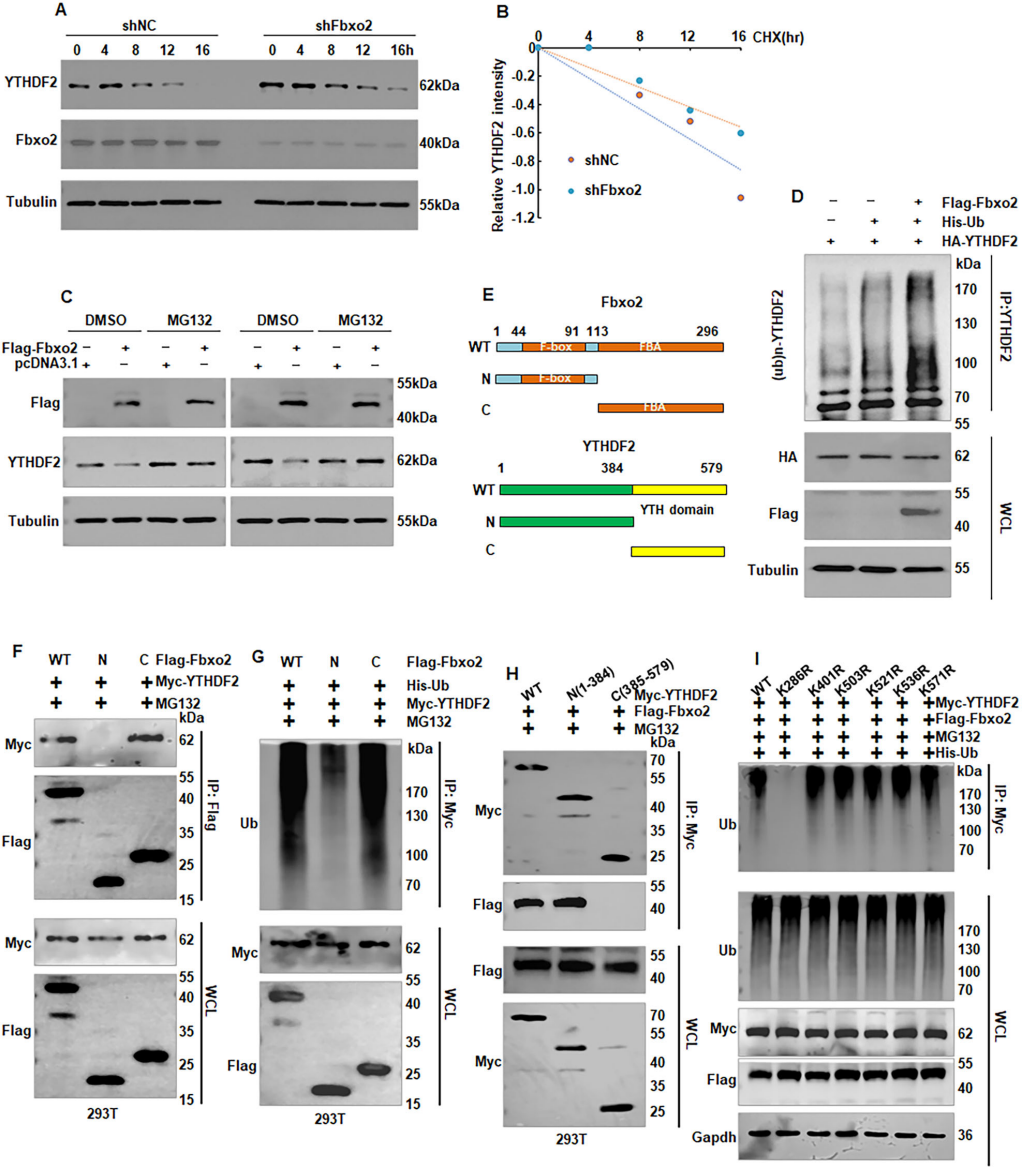
Fbxo2 mediates the ubiquitination of YTHDF2. **A** The protein half-life of YTHDF2 in DU145 with or without Fbxo2 depletion was identified by western blotting analysis. **B** Quantification of YTHDF2 protein levels from panel (**A**). **C** Treatment with MG132, a proteasome inhibitor, restores YTHDF2 protein levels reduced by Fbxo2 overexpression. **D** HEK293T cells were co-transfected with Flag-Fbxo2, His-Ub and Myc-YTHDF2 plasmids, followed by IP/IB assay as indicated. **E** Diagrammatic representation of Fbxo2 and YTHDF2 deletion mutants utilized for interaction and ubiquitination analysis. **F**, **G** HEK293T cells were co-transfected with His-Ub, Myc-YTHDF2, and either wild-type or mutant Flag-Fbxo2 plasmids. YTHDF2 was immunoprecipitated using an anti-Myc antibody, and ubiquitination was analyzed by Western blot. **H** HEK293T cells were transfected with the indicated plasmids and treated with 10 μM MG132 for 12 h, and subjected to IP and Western blot analysis. **I** HEK293T cells were co-transfected with His-Ub, Flag-Fbxo2, and Myc-YTHDF2 wild-type or lysine mutants (K286R, K401R, K503R, K521R, K536R, K571R). IP with anti-Myc was performed to identify the critical ubiquitination site of YTHDF2.

To investigate the mechanism by which Fbxo2 regulates YTHDF2 stability, we first performed cycloheximide (CHX) chase assays in DU145 and C4-2 cells. The results showed that Fbxo2 depletion extended the half-life of YTHDF2 protein (Fig. 5A, B, Supplementary Fig. S5A, B). Given that Fbxo2 is a component of the SCF E3 ubiquitin ligase complex, we next examined whether it regulates YTHDF2 through direct interaction. Overexpression of Fbxo2 in PC3 and DU145 cells led to a significant decrease in YTHDF2 protein levels. This reduction was completely reversed by treatment with the proteasome inhibitor MG132 (Fig. 5C). We further confirmed that Fbxo2 promotes the ubiquitination of YTHDF2. Ubiquitination assays in 293T cells indicated that exogenous Fbxo2 overexpression increased YTHDF2 ubiquitination (Fig. 5D). To map the interaction domains, we conducted domain-mapping experiments. We found that the carboxy-terminal (C-terminal) region of Fbxo2, rather than the amino terminal (N-terminal) domain, was required for binding to and mediating the ubiquitination and degradation of YTHDF2 (Fig. 5E–G). Conversely, the N-terminal domain (amino acids 1–384) of YTHDF2 was responsible for binding to Fbxo2 (Fig. 5H). Based on predictions from the GPS-Uber tool, we generated six lysine-to-arginine point mutations in YTHDF2 (K286R, K401R, K503R, K521R, K536R, and K571R) to identify potential ubiquitination sites. Ubiquitination assays demonstrated that the K286R mutations significantly impaired Fbxo2-mediated ubiquitination of YTHDF2, indicating that lysine 286 is a key ubiquitination site (Fig. 5I).

### YTHDF2 knockdown partially eliminates the enhanced proliferation and motility caused by Fbxo2 depletion

YTHDF2 is an essential m6A reader protein that typically facilitates the degradation of m6A-modified mRNAs [28]. Although it has been implicated in various cancers, its role in PCa remains unclear [29–31]. To address this, we assessed YTHDF2 expression and functions in PCa. IHC and western blot analysis revealed that YTHDF2 expression was markedly upregulated in PCa tissues and in three PCa cell lines (Supplementary Fig. S6A, B). To explore its function, we generated YTHDF2 knockdown models in PC3 and DU145 cells using two independent shRNAs (Supplementary Fig. S6C, D). CCK-8 assays (Supplementary Fig. S6E), colony formation assays (Supplementary Fig. S6F), and transwell assays (Supplementary Fig. S6G) indicated that downregulation of YTHDF2 restrained cell proliferation and invasion in PCa cells.

**Fig. 6:**
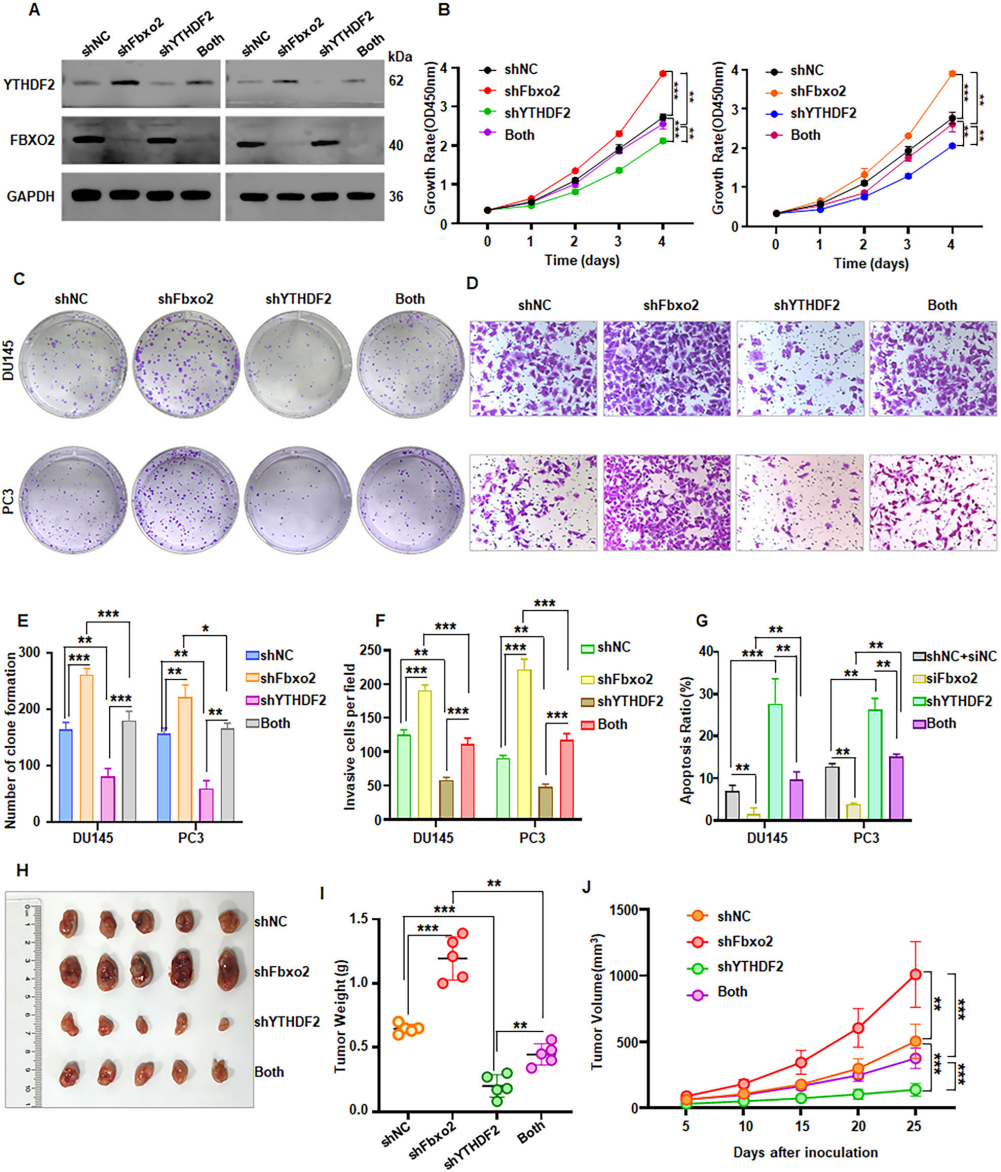
YTHDF2 knockdown partially eliminates the proliferative and invasive effects of Fbxo2 silencing in PCa. **A** Western blot analysis of Fbxo2 and YTHDF2 protein levels in PC3 and DU145 cells transduced with NC shRNA, shFbxo2, shYTHDF2, or shFbxo2+shYTHDF2 lentivirus. ShNC: NC shRNA; both: shFbxo2+shYTHDF2 lentivirus. CCK8 (**B**) and colony formation (**C**) tests were used to determine the cell viability and proliferation of PC3 and DU145 cells infected with NC shRNA, shFbxo2, shYTHDF2, or shFbxo2+shYTHDF2. **D** Transwell invasion assay showing that YTHDF2 knockdown partially eliminates the increased invasiveness caused by Fbxo2 silencing. Quantification of colony formation (**E**), invasion (**F**) and apoptosis (**G**). **H** Representative images of xenograft tumors generated by subcutaneous injection of DU145 cells infected with NC shRNA, shFbxo2, shYTHDF2, or shFbxo2+shYTHDF2. **I** Tumor weights from each group were displayed as the mean ± SEM. **J** Tumor volume measurements were taken every five days and tumor growth curves are shown. *p < 0.05, **p < 0.01, ***p < 0.001.

Next, in order to verify whether YTHDF2 is functionally involved in Fbxo2-mediated tumor suppression, we performed rescue experiments by co-transfecting cells with shFbxo2 and shYTHDF2 (Fig. 6A, Supplementary Fig. S7). Functional assays, including CCK-8, colony formation, transwell invasion, wound healing, and apoptosis analyses, revealed that the elevated oncogenic effects of Fbxo2 depletion were reversed by YTHDF2 knockdown (Fig. 6B–G, Supplementary Fig. S8A–C). Moreover, in vivo xenograft studies showed that the increased tumor volume and weight observed with Fbxo2 knockdown were reduced when YTHDF2 was simultaneously silenced (Fig. 6H–J). Furthermore, the YTHDF2 inhibitor DC-Y13-27, which has been reported to exhibit anti-tumor activity in breast cancer [32], was further evaluated in our prostate cancer cells. We found that DC-Y13-27 effectively suppressed the proliferation of PC3 and C4-2 cells, with an IC₅₀ of ~40 µM (Supplementary Fig. S9A). To elucidate its mechanism of action, we assessed the expression of relevant proteins by Western blotting and observed that treatment with 20 µM DC-Y13-27 markedly reduced YTHDF2 protein levels while concomitantly increasing CDKN1C protein expression (Supplementary Fig. S9B). Moreover, in FBXO2-knockdown cells, co-treatment with DC-Y13-27 significantly reversed the pro-proliferative effect induced by FBXO2 depletion (Supplementary Fig. S9C). These findings indicate that YTHDF2 is a key downstream effector in the Fbxo2-mediated suppression of PCa progression.

### YTHDF2 regulates the mRNA degradation of *CDKN1C* in an m^6^A-dependent manner

**Fig. 7:**
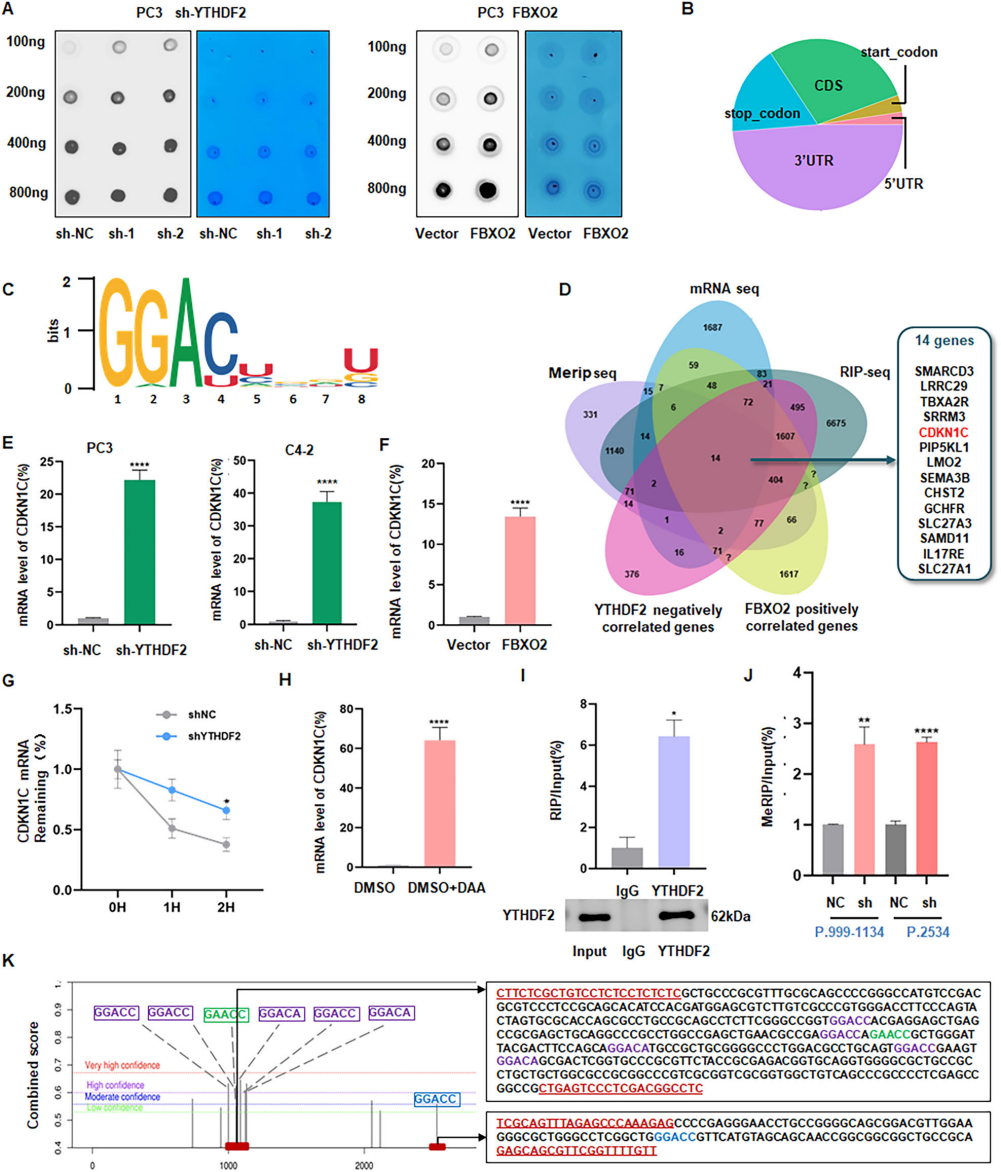
YTHDF2 promotes the degradation of *CDKN1C* mRNA in an m6A-dependent manner. **A** RNA dot blot assay was used to assess global m6A levels following Fbxo2 overexpression or knockdown. Methylene blue staining was used as the loading control. **B** Distribution of m6A modifications across mRNA transcripts, with enrichment primarily observed in the 3′ untranslated region (3′UTR). **C** Motif analysis using the program HOMER identified “GGACU”as the m6A consensus motif in PC3 cells. **D** Diagram integrating MeRIP-seq, Rip-seq, mRNA-seq, Fbxo2 positively correlated genes, and YTHDF2 negatively correlated genes. In total, 14 candidate genes were screened out. **E** Following YTHDF2 knockdown, the mRNA levels of CDKN1C were increased by RT-PCR analysis. **F** CDKN1C mRNA levels were increased in response to Fbxo2 overexpression by RT-qPCR analysis. **G** YTHDF2 knockdown extends the half-life of *CDKN1C* mRNA, as determined by RT-qPCR following treatment with actinomycin D (5 μg/mL) for the indicated time points. **H** The mRNA level of *CDKN1C* after 3-deazaadenosine (DAA) treatment was detect through RT-qPCR analysis. **I** RNA immunoprecipitation (RIP) test identifying the connection between YTHDF2 and *CDKN1C* mRNA in PC3 cells. **J** The m^6^A level alterations of *CDKN1C* after YTHDF2 knockdown were detected through MeRIP-RT-qPCR. **K** The probable m6A sites of *CDKN1C* were predicted by SRAMP. Purple color represents high-confidence sites. The sequences next to them are corresponding to the primers used in the groups with differences in the MeRIP-RT-qPCR experiments. *p < 0.05, **p < 0.01, ***p < 0.001.

It has already been illustrated that YTHDF2 could recognize m^6^A-modified sites and lead to the degradation of specific mRNAs [18]. In order to ascertain whether Fbxo2 regulates m^6^A enrichment through inhibiting YTHDF2, we conducted an RNA m^6^A dot-blot assay. In agreement with previous findings [22], YTHDF2 knockdown in prostate cancer cells results in increased global m^6^A levels. Conversely, Fbxo2 overexpression significantly increased overall m6A levels (Fig. 7A), indicating that Fbxo2 expression has a positive correlation with m^6^A methylation in PCa cells.

We then analyzed data from a previously published MeRIP-seq (m^6^A-seq) assay by Li et al. [22]. The results showed that the m^6^A peaks were predominantly located in the 3′ untranslated regions (3’UTRs, 48.7%), then the coding sequences (CDS, 28.7%), stop codon sites (17%), start codon sites (3.2%) and 5′ UTRs (2.4%) (Fig. 7B). Besides, the GGAC [U/A] motif was identified as the m6A consensus sequence in PC3 cells (Fig. 7C). Using the LinkedOmics database, we identified a compendium of genes positively correlated with Fbxo2 expression and negatively associated with YTHDF2 expression. A comprehensive integrative analysis of MeRIP-seq, mRNA-seq, RIP-seq data, and the Fbxo2/YTHDF2-associated gene lists revealed 14 overlapping candidate genes (Fig. 7D). Among these, *CDKN1C* emerged as a particularly interesting target, as it has been previously reported to function as a tumor suppressor in PCa [33, 34]. Further experiments demonstrated that YTHDF2 knockdown or Fbxo2 overexpression led to a substantial increase in *CDKN1C* mRNA expression (Fig. 7E, F). Moreover, the half-life of *CDKN1C* mRNA was prolonged in YTHDF2-depleted PC3 cells (Fig. 7G). Treatment with 3-deazaadenosine (DAA), a global methylation inhibitor, also resulted in a substantial rise in *CDKN1C* mRNA levels (Fig. 7H).

**Fig. 8:**
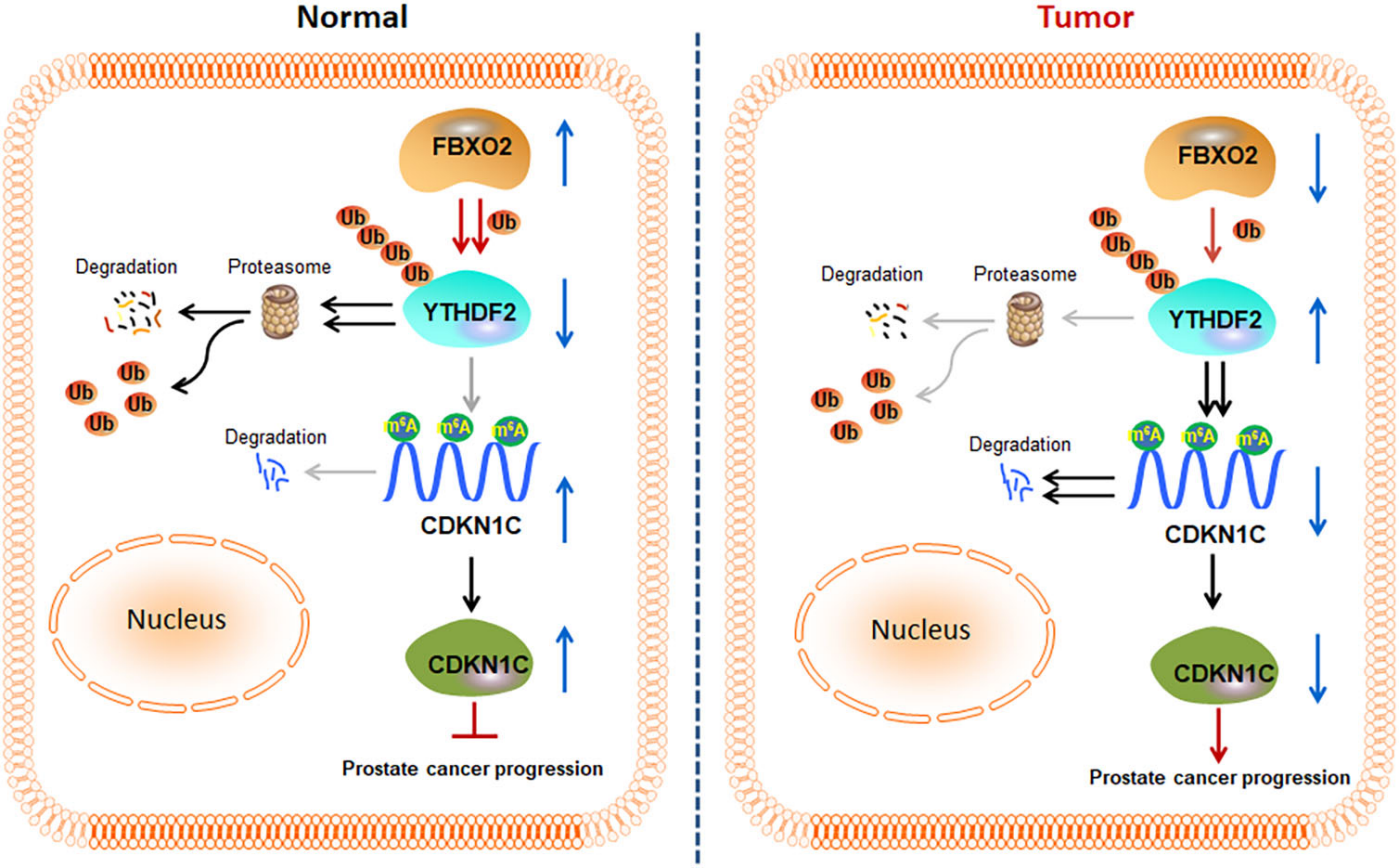
Schematic model of the Fbxo2-YTHDF2-CDKN1C cascade regulating m6A-dependent turnover of *CDKN1C* mRNA. Fbxo2 acts as an E3 ubiquitin ligase targeting YTHDF2 for proteasomal degradation, which in turn stabilizes CDKN1C mRNA by preventing its m6A-dependent decay. This regulatory cascade inhibits prostate cancer cell proliferation and progression.

To confirm the direct interaction between YTHDF2 and *CDKN1C* mRNA, RIP was performed in PC3 cells. The results showed that YTHDF2 greatly enriched the *CDKN1C* mRNA in comparison to the IgG control in PC3 cells (Fig. 7I). Subsequently, MeRIP-RT-qPCR assay was performed to verify these predicted sites. The results demonstrated enrichment of m^6^A-modified *CDKN1C* mRNA at two specific locations compared to the IgG control (Fig. 7J, K). In consideration of the findings, it can be posited that YTHDF2 exerts a regulatory influence on the mRNA degradation of *CDKN1C* by reading the specific m^6^A-modified sites in its 3’UTR. This regulatory mechanism may contribute to the tumor-suppressive function of the Fbxo2–YTHDF2–CDKN1C axis in prostate cancer (Fig. 8). We generated two CDKN1C 3′UTR m⁶A-site mutant plasmids in which the YTHDF2-binding motifs were disrupted, and compared them with the WT construct. Co-transfection experiments were performed in PC3 and C4-2 cells. The results clearly showed that YTHDF2 overexpression markedly reduced the protein level of WT CDKN1C, whereas the protein expression of the mutant CDKN1C constructs was not significantly affected by YTHDF2 overexpression (Supplementary Fig. S10).

We also demonstrated that CDKN1C expression was positively correlated with FBXO2 expression and negatively correlated with YTHDF2 expression in both subcutaneous xenograft tumors in nude mice and prostate cancer tissues (Supplementary Fig. S11A–D). To further confirm the role of the FBXO2–YTHDF2–CDKN1C axis in prostate cancer metastasis in vivo, control cells or FBXO2-overexpressing cells were injected into the lateral tail vein of nude mice. The results showed that the number of lung metastatic nodules was significantly reduced in the FBXO2-overexpression group compared with controls (Supplementary Fig. S12A–B). In addition, IHC analysis of metastatic nodules revealed that FBXO2 overexpression led to marked upregulation of CDKN1C and downregulation of YTHDF2 (Supplementary Fig. S12C). IHC staining of the human PCa tissue arrays shows that YTHDF2 expression is negatively correlated with Fbxo2 expression, and CDKN1C expression is positively correlated with Fbxo2 expression (Supplementary Fig. S12D). Moreover, high FBXO2 expression was significantly associated with better prognosis in PCa patients (Supplementary Fig. S12E). Taken together, these findings demonstrate that FBXO2 interacts with and promotes the degradation of the m6A reader YTHDF2 in prostate cancer, thereby inducing CDKN1C expression through an m6A-dependent mechanism.

## Discussion

Fbxo2 contributes to the formation of tumors; however, there are insufficient data to show its underlying mechanisms. Although some early evidence indicate that Fbxo2 inhibits tumor cell proliferation [34], more recent studies have reported its oncogenic role in several cancers by mediating the ubiquitination of key tumor suppressors such as p53, SUN2, and FBN1 [16, 35, 36]. In our study, Fbxo2 is considerably downregulated in PCa tissues compared to nearby normal tissues. Moreover, higher Fbxo2 expression is associated with improved patient prognosis. Functional assays, both in vitro and in vivo using a murine xenograft model, consistently showed that Fbxo2 overexpression inhibits PCa cell proliferation and motility. Collectively, our data support the identification of Fbxo2 as a tumor suppressor in prostate cancer.

As an E3 ubiquitin ligase, Fbxo2 facilitates substrate ubiquitination and destruction that is dependent on proteasomes [37]. While a few substrates of Fbxo2 have been previously indicated, our study uses co-IP-MS analysis to identify YTHDF2 as a novel substrate of Fbxo2. We further demonstrate that Fbxo2 promotes YTHDF2 ubiquitination and proteolytic degradation, providing a new mechanistic insight into Fbxo2-mediated tumor suppression. Since YTHDF2 is crucial for normal cellular development, dysregulation of YTHDF2 has been linked to various diseases, including cancer [38]. Importantly, multiple studies have suggested that YTHDF2 may function either as a tumor suppressor or an oncogene depending on the cancer type and cellular context [39]. For instance, YTHDF2 functions as a tumor suppressor in several cancers, including gastric cancer, hepatocellular carcinoma, and oral squamous cell carcinoma, by accelerating the decay of oncoproteins such as FOXC2, EGFR and eIF4G1 [40, 41]. On the other hand, YTHDF2 has also been shown to operate as an oncogenic factor by promoting the degradation of certain tumor suppressors, including TNFRSF1B, SETD7, and UBXN1 [42–44]. Consistent with previous report, we demonstrated that ablation of YTHDF2 inhibits cell proliferation and motility in PCa cell.

Given its dual role, recent studies have increasingly focused on understanding the regulatory mechanisms that control YTHDF2 protein levels. YTHDF2 expression is regulated at multiple levels, including transcriptional, post-transcriptional, and post-translational stages [27]. Recent studies have increasingly highlighted the diverse PTMs of YTHDF2 and their critical roles in cancer biology. For instance, SUMOylation of YTHDF2 at lysine 571 markedly enhances its binding affinity for m6A-modified transcripts, thereby promoting tumor growth [45]. In addition, EGFR-mediated phosphorylation at serine 39 and threonine 381 stabilizes YTHDF2 and accelerates the degradation of HIVEP2 and LXRA mRNAs, contributing to glioblastoma progression [21]. Furthermore, acetylation of lysine 542 (K542), dynamically regulated by p300 and SIRT2, strengthens YTHDF2–m6A interactions and facilitates colorectal cancer development. Although FBXW7 and SKP2 have been identified as E3 ubiquitin ligases that target YTHDF2, and OTUB1 as a deubiquitinase that stabilizes it [25–27], the specific ubiquitination regions and lysine residues involved remained unclear. Our findings clarify this by showing that Fbxo2 binds to the N-terminal domain (amino acids 1–384) of YTHDF2 and promotes its ubiquitination at lysine 286 (K286). Notably, YTHDF2 ubiquitination appears to be differentially regulated by distinct E3 ligases. FBXW7 and Skp2 promote YTHDF2 degradation in a phosphorylation-dependent manner during cell cycle progression, whereas Fbxo2 mediates its turnover in response to metabolic stress through O-GlcNAcylation [25–27]. Further studies are required to precisely define the degradation signals involved.

Different types of cancer appear to have distinct downstream effectors of YTHDF2. In this study, we identified *CDKN1C* as a crucial effector of YTHDF2 in PCa cells. *CDKN1C* gene encodes p57^Kip2^ protein and it belongs to the CIP/Kip family of cyclin-dependent kinase inhibitors, which also includes p21^Cip1^ and p27^Kip1^ [46, 47]. The tumor suppressive potential of *CDKN1C* is highlighted by its association with sporadic cancers and Beckwith–Wiedemann syndrome when mutated or deleted [48]. Furthermore, it has been discovered that p57^Kip2^-deficient animals display immaturity of germinal tissues, including the ovaries, uterus, testes, and prostate, underscoring the critical function of p57^Kip2^ in the development of reproductive system [49]. Moreover, a poor prognosis is linked to decreased p57 expression, which is correlated with aggressiveness in a variety of tumor types [50]. Downregulation of *CDKN1C* is typically observed in multiple malignancies, including breast cancer [51], leukemias [52], adrenocortical [53], as well as urothelial cancer [54], PCa [33]. Hence, YTHDF2-mediated degradation of CDKN1C contributes to tumor progression, and support the hypothesis that restoring CDKN1C expression may serve as a therapeutic strategy in prostate cancer.

In summary, our study reveals a previously underappreciated regulatory axis—Fbxo2/YTHDF2/CDKN1C—that plays a critical role in PCa progression. Fbxo2 acts as a tumor suppressor by targeting the oncogenic m6A reader YTHDF2 for ubiquitination and proteasomal degradation, thereby stabilizing m6A-modified tumor-suppressive mRNAs such as CDKN1C. This stabilization inhibits the survival and proliferation of PCa cells. Our findings not only provide new insights into the molecular mechanisms underlying PCa but also highlight the therapeutic potential of targeting the Fbxo2–YTHDF2–CDKN1C axis for future anticancer strategies.

## Materials and methods

### Cell culture

The human embryonic kidney cell line 293T and PCa cell lines PC3, DU145, and 22RV1 were purchased from Procell Life Science & Technology Co., Ltd. (Wuhan, China). DU145 cells were cultured in minimum essential medium (MEM; Gibco), PC3 cells in Ham’s F-12K medium (Procell, China), and 22RV1 in RPMI-1640 medium (Gibco). All media were supplemented with 10% fetal bovine serum (FuHeng, Shanghai, China), and cells were maintained at 37 °C in 5% CO_2_ environment. The non-tumorigenic human prostate epithelial cell line RWPE-1 was acquired from FuHeng Cell Center (FuHeng, Shanghai, China) and cultured in Keratinocyte serum-free medium (K-SFM) supplemented with 5 ng/ml human recombinant epidermal growth factor (EGF) and 0.05 mg/ml bovine pituitary extract (BPE) at 37 °C in 5% CO_2_ environment.

### Establishment of stable cell lines

Plasmids encoding shRNAs (Supplementary Table S1), siRNAs (Supplementary Table S2), Fbxo2, or empty vector controls were co-transfected into HEK293T cells along with packaging plasmids (pSPAX2 and pMD2G). Lentivral supernatants were collected and filtered through a 0.45 µm filter after 48-h post-transfection. PCa cells were infected with the lentiviral particles in the presence of 10 μg/mL Polybrene (GenePharma, Shanghai, China). After 48 h, cells were selected with 2 μg/mL puromycin for three days. The efficiency of gene overexpression or knockdown was verified via RT-qPCR and western blotting.

### Colony formation and CCK-8 cell viability assays

Colony formation and cell counting kit-8 (CCK-8) assays were performed to evaluate in vitro cell proliferation, following protocols described in previous studies [55].

### Apoptosis assay

As directed by the manufacturer, the Annexin V-FITC/PI apoptosis kit (Multisciences, China) and FACS were utilized for the cell apoptosis assay. In brief, cells were harvested, washed twice with cold PBS, and resuspended in 500 μL of 1×binding buffer. Then, 5 µL of Annexin V-FITC and 10 μL of propidium iodide (PI) were added to the suspension. Apoptotic cells were analyzed by flow cytometry (Beckman, Germany).

### Wound healing assay

The migratory ability of PCa cells was evaluated using a wound healing assay. Cells were seeded into 6-well plates and cultured and allowed to develop complete confluence. A sterile 200 µL yellow pipette tip was used to create a linear scratch through the cell monolayer. Detached cells and debris were removed by washing twice with sterile PBS. The cells were then cultured for 20 h in serum-free medium. Images of the wound area were captured at 0 and 20 h using an optical microscope. The wound closure was quantified using ImageJ software.

### Western blotting

Total protein was extracted and separated by SDS-PAGE, followed by transfer onto a nitrocellulose (NC) membrane. The membrane was blocked with 5% skim milk for 1 h at room temperature and then incubated with primary antibodies, followed by the corresponding secondary antibodies. Protein bands were visualized using Omni-ECL™ reagent (YEASEN, Shanghai, China) [56]. The primary antibodies included anti-Fbxo2 (14590-1-AP, Proteintech), anti-Myc (16286-1-AP, Proteintech), anti-HA (51064-2-AP, Proteintech), anti-Flag (F1804, Sigma), anti-YTHDF2 (#71283, CST), anti-GAPDH (GB15004-100, Servicebio), anti-Actin(GB15003-100, Servicebio), and anti-Ub (sc-166553, Santa Cruz Biotechnology).

### RNA extraction and RT-qPCR

Total RNA was extracted from cell lines using TRIzol reagent (Invitrogen). Reverse transcription was performed using the RevertAid RT Kit (Thermo Fisher Scientific, USA). Quantitative PCR (qPCR) was conducted using SYBR Green Mix (Novoprotein, China). Primers for Fbxo2, YTHDF2 and *CDKN1C* (Supplementary Table S3) were purchased from Sangon Biotech Company (Shanghai, China).

### Transwell invasion assay

Cell invasion ability was assessed using Transwell chambers (Corning, USA) pre-coated with Matrigel (1:8 dilution in serum-free medium, Corning, USA) for 30 min at 37 °C. The lower chambers were supplied with medium containing 10% FBS as a chemoattractant. Then, 3 × 10^4^ cells were suspended in serum-free medium and seeded into the upper chambers. After a 24-h incubation, non-invading cells and residual Matrigel were removed from the upper side of the membrane using a cotton swab. Invaded cells on the underside were then fixed, stained, and imaged.

### Co-immunoprecipitation (co-IP)

Cells were harvested and lysed in NP-40 PIPA buffer for 30 min on ice. The protein lysates were incubated overnight at 4 °C with 5 μg of the appropriate primary antibody. Subsequently, 50 μl of Protein A/G magnetic beads (B23202, Selleck) were added to the mixture and incubated for 4 h at 4 °C. The beads were then washed with lysis buffer to remove non-specific proteins, and the immunoprecipitates were analyzed by western blotting [57].

### In vivo ubiquitination assay

Plasmids encoding Flag-tagged Fbxo2 (wild-type or deletion mutant), Myc-tagged YTHDF2-WT, and His-tagged ubiquitin were co-transfected into 293T cells. After 38 h of transfection, the cells were exposed to 10 μM MG132 for additional 10 h to inhibit proteasomal degradation. Cells were then harvested and lysed in NP-40 lysis buffer containing protease inhibitors. Lysates were incubated overnight at 4 °C with an anti-Myc antibody, following by a 4-h incubation with Protein A/G magnetic beads (B23202 Selleck). Ubiquitinated YTHDF2 was subsequently eluted and identified by Western blotting.

### Immunofluorescence

DU145 and PC3 cells were seeded onto 24-well dishes, fixed with 4% paraformaldehyde for 20 min, and permeabilized with 0.5% Triton X-100 for another 20 min. After blocking with 1% BSA for 30 min, cells were treated for overnight at 4 °C with anti-Fbxo2 and anti-YTHDF2 antibodies. The following day, cells were incubated with fluorescently labeled secondary antibodies for 1 h at room temperature. Nuclei were counterstained with DAPI. Fluorescence images were captured using a Zeiss confocal microscope.

### In vivo nude mice xenograft studies and cancer metastasis model

All animal procedures were approved by the Institutional Animal Care and Use Committee of Soochow University. Six-week-old male BALB/c nude mice (SLAC Laboratory Animal Company, Shanghai, China) were used for xenograft experiments. DU145 cells (5 × 10^6^ in 10 μl PBS) infected with shFbxo2, shYTHDF2 or shFbxo2+shYTHDF2 were injected subcutaneously into the flanks of mice (*n* = 5 per group). Tumor volume was measured every five days using the formula: V = L × W^2^ × 0.52 (L: the longest diameter; W: the shortest diameter). After 25 days, mice were sacrificed, and tumors were excised and weighed. Following the approved procedures of Soochow University’s Institutional Animal Care and Use Committee (IACUC), the lung cancer cell metastasis model was carried out. In 6-week-old male nude mice, 3 × 10^6^ PC3 cells overexpressing either NC or FBXO2 were injected into the lateral tail vein. After 6 weeks, the mice were sacrificed and their lungs were collected and analyzed by H&E staining.

### m6A quantification

To quantify total m6A levels, an RNA dot blot assay was carried out. Total RNA was isolated from cells and denatured at 95 °C for 5 min using a PCR thermal cycler. Equal amounts of RNA were spotted onto a NC membrane and crosslinked under 302 nm UV light for 30 min. The membrane was blocked with 5% skim milk for 2 h, followed by incubation overnight at 4 °C with an anti-m6A antibody (Abcam, UK). An ECL kit (Epizyme, China) was used to visualize the membrane after it was incubated with a secondary antibody. A parallel membrane was stained with methylene blue for 10 min in order to verify equal RNA loading and allow imaging.

### m6A-RNA immunoprecipitation (MeRIP) assay

RNA was isolated from shNC or shFbxo2 infected cells and sonicated into fragments. Samples were then incubated with m6A antibody and protein A/G beads sequentially by using a BersinBio™ m6A MeRIP Kit (Guangzhou, China) to perform the immunoprecipitation and purification. Then, mRNA modified by m6A was detected through qRT-PCR.

### Lentivirus transfection of organoids

Matrix gel was first added to the 24-well plate and allowed to solidify as a base layer. After collecting organoids by centrifugation, they were carefully extracted from the matrix gel and exposed to lentivirus. To enhance infection efficiency, a co-transfection reagent was added after resuspending the organoids in complete medium that contained a certain quantity of virus particles, and this mixture was then transferred to the pre-coated plates. Sixteen hours later, the medium containing the viral inoculum and floating organoids was aspirated, and the remaining organoids were overlaid with a fresh layer of matrix gel, allowed to solidify, and the covered with complete medium for further incubation. Western blotting was performed to verify transfection efficiency.

### Statistical analysis

All statistical analyses were conducted by GraphPad prism 8.0 or SPSS Statistics. Our data were presented as the mean ± S.D. Student’s *t* test was used to compare the differences between two different groups. Meanwhile, we examined statistical associations among the multiple groups by the ANOVA. Statistical significance was defined as a *p*-value less than 0.05.

## Supplementary information

Supplementary figures

Supplementary tables

Original Western blotting images

## Data Availability

The data are available upon reasonable request.

## References

[1] Almeeri MNE, Awies M, Constantinou C. Prostate cancer, pathophysiology and recent developments in management: a narrative review. Curr Oncol Rep. 2024;26:1511–9. 39453576 10.1007/s11912-024-01614-6

[2] Siegel RL, Giaquinto AN, Jemal A. Cancer statistics, 2024. CA Cancer J Clin. 2024;74:12–49. 38230766 10.3322/caac.21820

[3] Xiao J, Zhang M, Wu D. Side effects of prostate cancer therapies and potential management. J Biol Methods. 2024;11:e99010018. 39544189 10.14440/jbm.2024.0019 PMC11557297

[4] Zi H, Liu MY, Luo LS, Huang Q, Luo PC, Luan HH, et al. Global burden of benign prostatic hyperplasia, urinary tract infections, urolithiasis, bladder cancer, kidney cancer, and prostate cancer from 1990 to 2021. Mil Med Res. 2024;11:64. 39294748 10.1186/s40779-024-00569-w PMC11409598

[5] Feng DC, Zhu WZ, Wang J, Li DX, Shi X, Xiong Q, et al. The implications of single-cell RNA-seq analysis in prostate cancer: unraveling tumor heterogeneity, therapeutic implications and pathways towards personalized therapy. Mil Med Res. 2024;11:21. 38605399 10.1186/s40779-024-00526-7 PMC11007901

[6] Siegel DA, O’Neil ME, Richards TB, Dowling NF, Weir HK. Prostate cancer incidence and survival, by stage and race/ethnicity - United States, 2001–17. MMWR Morb Mortal Wkly Rep. 2020;69:1473–80. 33056955 10.15585/mmwr.mm6941a1 PMC7561091

[7] Tekcham DS, Chen D, Liu Y, Ling T, Zhang Y, Chen H, et al. F-box proteins and cancer: an update from functional and regulatory mechanism to therapeutic clinical prospects. Theranostics. 2020;10:4150–67. 32226545 10.7150/thno.42735 PMC7086354

[8] Cockram PE, Kist M, Prakash S, Chen SH, Wertz IE, Vucic D. Ubiquitination in the regulation of inflammatory cell death and cancer. Cell Death Differ. 2021;28:591–605. 33432113 10.1038/s41418-020-00708-5 PMC7798376

[9] Cai C, Tang YD, Zhai J, Zheng C. The RING finger protein family in health and disease. Signal Transduct Target Ther. 2022;7:300. 36042206 10.1038/s41392-022-01152-2 PMC9424811

[10] Xiong HJ, Yu HQ, Zhang J, Fang L, Wu D, Lin XT, et al. Elevated FBXL6 activates both wild-type KRAS and mutant KRAS(G12D) and drives HCC tumorigenesis via the ERK/mTOR/PRELID2/ROS axis in mice. Mil Med Res. 2023;10:68. 38124228 10.1186/s40779-023-00501-8 PMC10731709

[11] Heo J, Eki R, Abbas T. Deregulation of F-box proteins and its consequence on cancer development, progression and metastasis. Semin Cancer Biol. 2016;36:33–51. 26432751 10.1016/j.semcancer.2015.09.015 PMC4761475

[12] Huang Y, Che X, Wang PW, Qu X. p53/MDM2 signaling pathway in aging, senescence and tumorigenesis. Semin Cancer Biol. 2024;101:44–57. 38762096 10.1016/j.semcancer.2024.05.001

[13] Wang Z, Liu P, Inuzuka H, Wei W. Roles of F-box proteins in cancer. Nat Rev Cancer. 2014;14:233–47. 24658274 10.1038/nrc3700 PMC4306233

[14] Nishio K, Yoshida Y, Tanaka K, Mizushima T. Structural analysis of a function-associated loop mutant of the substrate-recognition domain of Fbs1 ubiquitin ligase. Acta Crystallogr F Struct Biol Commun. 2016;72:619–26. 27487926 10.1107/S2053230X16011018 PMC4973303

[15] Zhao X, Guo W, Zou L, Hu B. FBXO2 modulates STAT3 signaling to regulate proliferation and tumorigenicity of osteosarcoma cells. Cancer Cell Int. 2020;20:245. 32549792 10.1186/s12935-020-01326-4 PMC7296666

[16] Ji J, Shen J, Xu Y, Xie M, Qian Q, Qiu T, et al. FBXO2 targets glycosylated SUN2 for ubiquitination and degradation to promote ovarian cancer development. Cell Death Dis. 2022;13:442. 10.1038/s41419-022-04892-9 PMC9079088 35525855

[17] Buehler M, Yi X, Ge W, Blattmann P, Rushing E, Reifenberger G, et al. Quantitative proteomic landscapes of primary and recurrent glioblastoma reveal a protumorigeneic role for FBXO2-dependent glioma-microenvironment interactions. Neuro Oncol. 2023;25:290–302. 35802605 10.1093/neuonc/noac169 PMC9925714

[18] Wang X, Lu Z, Gomez A, Hon GC, Yue Y, Han D, et al. N6-methyladenosine-dependent regulation of messenger RNA stability. Nature. 2014;505:117–20. 24284625 10.1038/nature12730 PMC3877715

[19] Deng X, Su R, Weng H, Huang H, Li Z, Chen J. RNA N(6)-methyladenosine modification in cancers: current status and perspectives. Cell Res. 2018;28:507–17. 29686311 10.1038/s41422-018-0034-6 PMC5951805

[20] Einstein JM, Perelis M, Chaim IA, Meena JK, Nussbacher JK, Tankka AT, et al. Inhibition of YTHDF2 triggers proteotoxic cell death in MYC-driven breast cancer. Mol Cell. 2021;81:3048–64.e3049. 34216543 10.1016/j.molcel.2021.06.014 PMC8359670

[21] Fang R, Chen X, Zhang S, Shi H, Ye Y, Shi H, et al. EGFR/SRC/ERK-stabilized YTHDF2 promotes cholesterol dysregulation and invasive growth of glioblastoma. Nat Commun. 2021;12:177. 33420027 10.1038/s41467-020-20379-7 PMC7794382

[22] Li J, Xie H, Ying Y, Chen H, Yan H, He L, et al. YTHDF2 mediates the mRNA degradation of the tumor suppressors to induce AKT phosphorylation in N6-methyladenosine-dependent way in prostate cancer. Mol Cancer. 2020;19:152. 33121495 10.1186/s12943-020-01267-6 PMC7599101

[23] Hou J, Zhang H, Liu J, Zhao Z, Wang J, Lu Z, et al. YTHDF2 reduction fuels inflammation and vascular abnormalization in hepatocellular carcinoma. Mol Cancer. 2019;18:163. 31735169 10.1186/s12943-019-1082-3 PMC6859620

[24] Zhong L, Liao D, Zhang M, Zeng C, Li X, Zhang R, et al. YTHDF2 suppresses cell proliferation and growth via destabilizing the EGFR mRNA in hepatocellular carcinoma. Cancer Lett. 2019;442:252–61. 30423408 10.1016/j.canlet.2018.11.006

[25] Fei Q, Zou Z, Roundtree IA, Sun HL, He C. YTHDF2 promotes mitotic entry and is regulated by cell cycle mediators. PLoS Biol. 2020;18:e3000664. 32267835 10.1371/journal.pbio.3000664 PMC7170294

[26] Xu F, Li J, Ni M, Cheng J, Zhao H, Wang S, et al. FBW7 suppresses ovarian cancer development by targeting the N(6)-methyladenosine binding protein YTHDF2. Mol Cancer. 2021;20:45. 33658012 10.1186/s12943-021-01340-8 PMC7927415

[27] Zhao X, Lv S, Li N, Zou Q, Sun L, Song T. YTHDF2 protein stabilization by the deubiquitinase OTUB1 promotes prostate cancer cell proliferation via PRSS8 mRNA degradation. J Biol Chem. 2024;300:107152. 38462165 10.1016/j.jbc.2024.107152 PMC11002313

[28] Chen X, Zhou X, Wang X. m(6)A binding protein YTHDF2 in cancer. Exp Hematol Oncol. 2022;11:21. 35382893 10.1186/s40164-022-00269-y PMC8981655

[29] Bai X, Chen J, Zhang W, Zhou S, Dong L, Huang J, et al. YTHDF2 promotes gallbladder cancer progression and gemcitabine resistance via m6A-dependent DAPK3 degradation. Cancer Sci. 2023;114:4299–313. 37700438 10.1111/cas.15953 PMC10637062

[30] Zhang L, Li Y, Zhou L, Zhou H, Ye L, Ou T, et al. The m6A reader YTHDF2 promotes bladder cancer progression by suppressing RIG-I-mediated immune response. Cancer Res. 2023;83:1834–50. 36939388 10.1158/0008-5472.CAN-22-2485 PMC10236158

[31] Zhang C, Huang S, Zhuang H, Ruan S, Zhou Z, Huang K, et al. YTHDF2 promotes the liver cancer stem cell phenotype and cancer metastasis by regulating OCT4 expression via m6A RNA methylation. Oncogene. 2020;39:4507–18. 32366907 10.1038/s41388-020-1303-7

[32] Shuai Y, Ma Z, Ju J, Li C, Bai X, Yue J, et al. The N6-methyladenosine writer METTL3 promotes breast cancer progression through YTHDF2-dependent posttranscriptional silencing of GSDMD. Apoptosis. 2025;30:226–38. 39627574 10.1007/s10495-024-02037-1

[33] Jin RJ, Lho Y, Wang Y, Ao M, Revelo MP, Hayward SW, et al. Down-regulation of p57Kip2 induces prostate cancer in the mouse. Cancer Res. 2008;68:3601–8. 18483241 10.1158/0008-5472.CAN-08-0073

[34] Erhardt JA, Hynicka W, DiBenedetto A, Shen N, Stone N, Paulson H, et al. A novel F box protein, NFB42, is highly enriched in neurons and induces growth arrest. J Biol Chem. 1998;273:35222–7. 9857061 10.1074/jbc.273.52.35222

[35] Che X, Jian F, Wang Y, Zhang J, Shen J, Cheng Q, et al. FBXO2 promotes proliferation of endometrial cancer by ubiquitin-mediated degradation of FBN1 in the regulation of the cell cycle and the autophagy pathway. Front Cell Dev Biol. 2020;8:843. 32984335 10.3389/fcell.2020.00843 PMC7487413

[36] Guo W, Ren Y, Qiu X. FBXO2 promotes the progression of papillary thyroid carcinoma through the p53 pathway. Sci Rep. 2024;14:22574. 39343799 10.1038/s41598-024-73455-z PMC11439943

[37] Zhang HJ, Tian J, Qi XK, Xiang T, He GP, Zhang H, et al. Epstein-Barr virus activates F-box protein FBXO2 to limit viral infectivity by targeting glycoprotein B for degradation. PLoS Pathog. 2018;14:e1007208. 30052682 10.1371/journal.ppat.1007208 PMC6082576

[38] Wang JY, Lu AQ. The biological function of m6A reader YTHDF2 and its role in human disease. Cancer Cell Int. 2021;21:109. 33593354 10.1186/s12935-021-01807-0 PMC7885220

[39] Liu R, Jia Y, Kong G, He A. Novel insights into roles of N6-methyladenosine reader YTHDF2 in cancer progression. J Cancer Res Clin Oncol. 2022;148:2215–30. 35763107 10.1007/s00432-022-04134-7 PMC11800943

[40] Shen X, Zhao K, Xu L, Cheng G, Zhu J, Gan L, et al. YTHDF2 inhibits gastric cancer cell growth by regulating FOXC2 signaling pathway. Front Genet. 2021;11:592042. 33505426 10.3389/fgene.2020.592042 PMC7831514

[41] Wang F, Liao Y, Zhang M, Zhu Y, Wang W, Cai H, et al. N6-methyladenosine demethyltransferase FTO-mediated autophagy in malignant development of oral squamous cell carcinoma. Oncogene. 2021;40:3885–98. 33972683 10.1038/s41388-021-01820-7

[42] Chai RC, Chang YZ, Chang X, Pang B, An SY, Zhang KN, et al. YTHDF2 facilitates UBXN1 mRNA decay by recognizing METTL3-mediated m(6)A modification to activate NF-kappaB and promote the malignant progression of glioma. J Hematol Oncol. 2021;14:109. 34246306 10.1186/s13045-021-01124-z PMC8272379

[43] Chen Z, Shao YL, Wang LL, Lin J, Zhang JB, Ding Y, et al. YTHDF2 is a potential target of AML1/ETO-HIF1alpha loop-mediated cell proliferation in t(8;21) AML. Oncogene. 2021;40:3786–98. 33958724 10.1038/s41388-021-01818-1

[44] Xie H, Li J, Ying Y, Yan H, Jin K, Ma X, et al. METTL3/YTHDF2 m(6) A axis promotes tumorigenesis by degrading SETD7 and KLF4 mRNAs in bladder cancer. J Cell Mol Med. 2020;24:4092–104. 32126149 10.1111/jcmm.15063 PMC7171394

[45] Hou G, Zhao X, Li L, Yang Q, Liu X, Huang C, et al. SUMOylation of YTHDF2 promotes mRNA degradation and cancer progression by increasing its binding affinity with m6A-modified mRNAs. Nucleic Acids Res. 2021;49:2859–77. 33577677 10.1093/nar/gkab065 PMC7969013

[46] Lee MH, Reynisdottir I, Massague J. Cloning of p57KIP2, a cyclin-dependent kinase inhibitor with unique domain structure and tissue distribution. Genes Dev. 1995;9:639–49. 7729683 10.1101/gad.9.6.639

[47] Matsuoka S, Edwards MC, Bai C, Parker S, Zhang P, Baldini A, et al. p57KIP2, a structurally distinct member of the p21CIP1 Cdk inhibitor family, is a candidate tumor suppressor gene. Genes Dev. 1995;9:650–62. 7729684 10.1101/gad.9.6.650

[48] Creff J, Besson A. Functional Versatility of the CDK Inhibitor p57(Kip2). Front Cell Dev Biol. 2020;8:584590. 33117811 10.3389/fcell.2020.584590 PMC7575724

[49] Takahashi K, Nakayama K, Nakayama K. Mice lacking a CDK inhibitor, p57Kip2, exhibit skeletal abnormalities and growth retardation. J Biochem. 2000;127:73–83. 10731669 10.1093/oxfordjournals.jbchem.a022586

[50] Kavanagh E, Joseph B. The hallmarks of CDKN1C (p57, KIP2) in cancer. Biochim Biophys Acta. 2011;1816:50–56. 21447370 10.1016/j.bbcan.2011.03.002

[51] Qiu Z, Li Y, Zeng B, Guan X, Li H. Downregulated CDKN1C/p57(kip2) drives tumorigenesis and associates with poor overall survival in breast cancer. Biochem Biophys Res Commun. 2018;497:187–93. 29428729 10.1016/j.bbrc.2018.02.052

[52] Radujkovic A, Dietrich S, Andrulis M, Benner A, Longerich T, Pellagatti A, et al. Expression of CDKN1C in the bone marrow of patients with myelodysplastic syndrome and secondary acute myeloid leukemia is associated with poor survival after conventional chemotherapy. Int J Cancer. 2016;139:1402–13. 27170453 10.1002/ijc.30181

[53] Giovannoni I, Boldrini R, Benedetti MC, Inserra A, De Pasquale MD, Francalanci P. Pediatric adrenocortical neoplasms: immunohistochemical expression of p57 identifies loss of heterozygosity and abnormal imprinting of the 11p15.5. Pediatr Res. 2017;81:468–72. 27842055 10.1038/pr.2016.239

[54] Hoffmann MJ, Florl AR, Seifert HH, Schulz WA. Multiple mechanisms downregulate CDKN1C in human bladder cancer. Int J Cancer. 2005;114:406–13. 15551363 10.1002/ijc.20749

[55] Yang C, Xiang H, Fu K, Jin L, Yuan F, Xue B, et al. Lycorine suppresses cell growth and invasion via down-regulation of NEDD4 ligase in bladder cancer. Am J Cancer Res. 2022;12:4708–20. 36381314 PMC9641406

[56] Weng H, Xiong KP, Wang W, Qian KY, Yuan S, Wang G, et al. Aspartoacylase suppresses prostate cancer progression by blocking LYN activation. Mil Med Res. 2023;10:25. 37271807 10.1186/s40779-023-00460-0 PMC10240701

[57] Xie MX, Lai RC, Xiao YB, Zhang X, Cao XY, Tian XY, et al. Endophilin A2 controls touch and mechanical allodynia via kinesin-mediated Piezo2 trafficking. Mil Med Res. 2024;11:17. 38475827 10.1186/s40779-024-00520-z PMC10929226

