# Supplementary material for: Fbxo2 suppresses prostate cancer progression by regulating YTHDF2 ubiquitination and degradation
Source: Cell Death Dis. 2025 Dec 29;17(1):153. doi: 10.1038/s41419-025-08396-0 (PMC12858993; doi:10.1038/s41419-025-08396-0)
Supplement: Supplementary file 1 — Supplementary figures [file 41419_2025_8396_MOESM1_ESM.pdf]

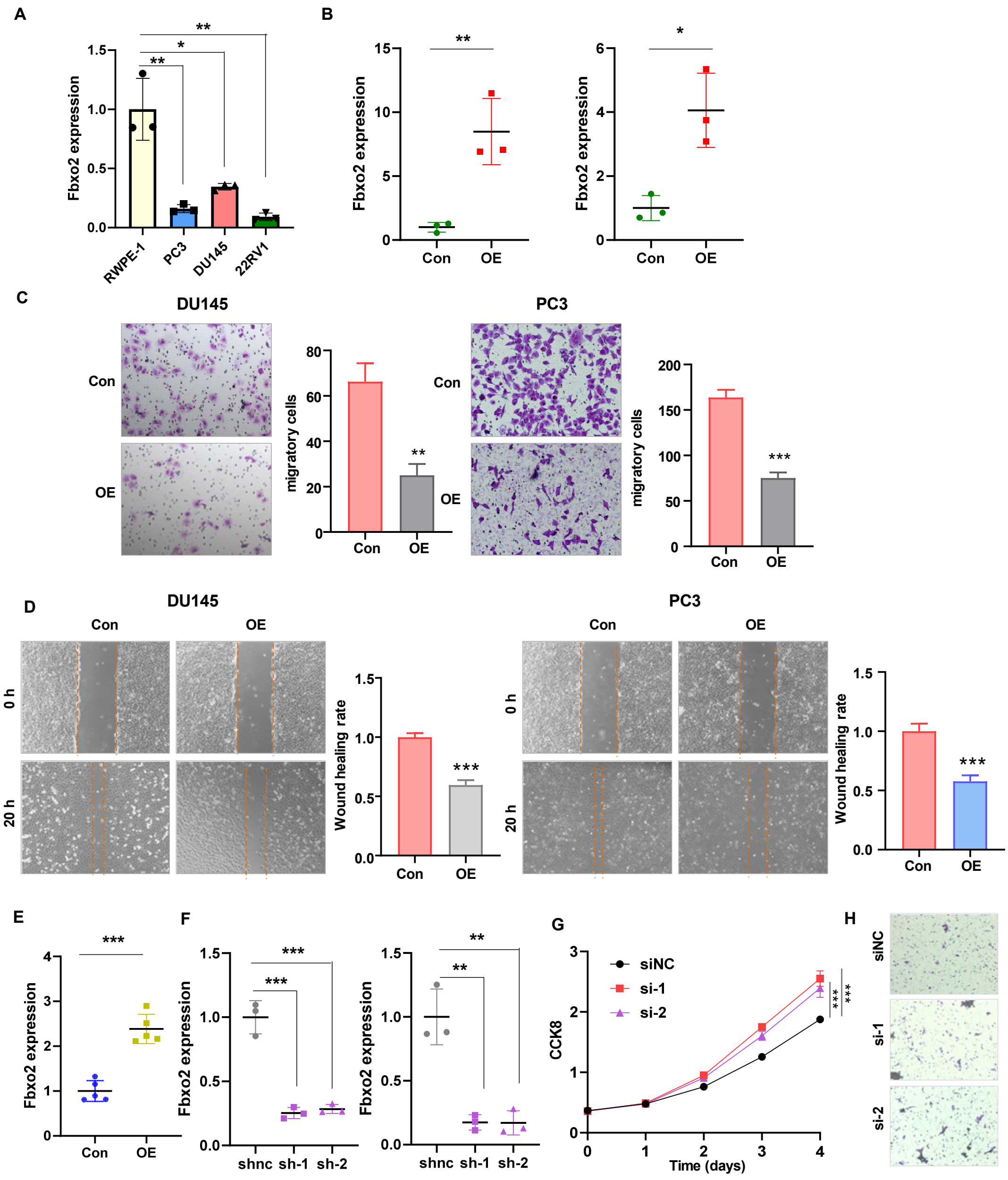

Fig. S1 Overexpression of Fbxo2 inhibits cell migration in PCa.

A Quantitative analysis of Fbxo2 protein levels in Figure 1G.

B Quantitative analysis of Fbxo2 protein levels in Figure 2A.

C-D In order to identify cell migration ability, the wound-healing experiment and the Transwell (without Matrigel) assay were applied to PC3 and DU145 cells, either with or without Fbxo2 overexpression.

E Quantitative analysis of Fbxo2 protein levels in Figure 2I.

F Quantitative analysis of Fbxo2 protein levels in Figure 3A. *p < 0.05, **p < 0.01, ***p < 0.001.

G Cell viability was assessed by CCK-8 assay in Fbxo2-knockdown C4-2 cells.

H The invasion capability of C4-2 cells treated with NC siRNA or Fbxo2 siRNA was evaluated by Transwell assay.

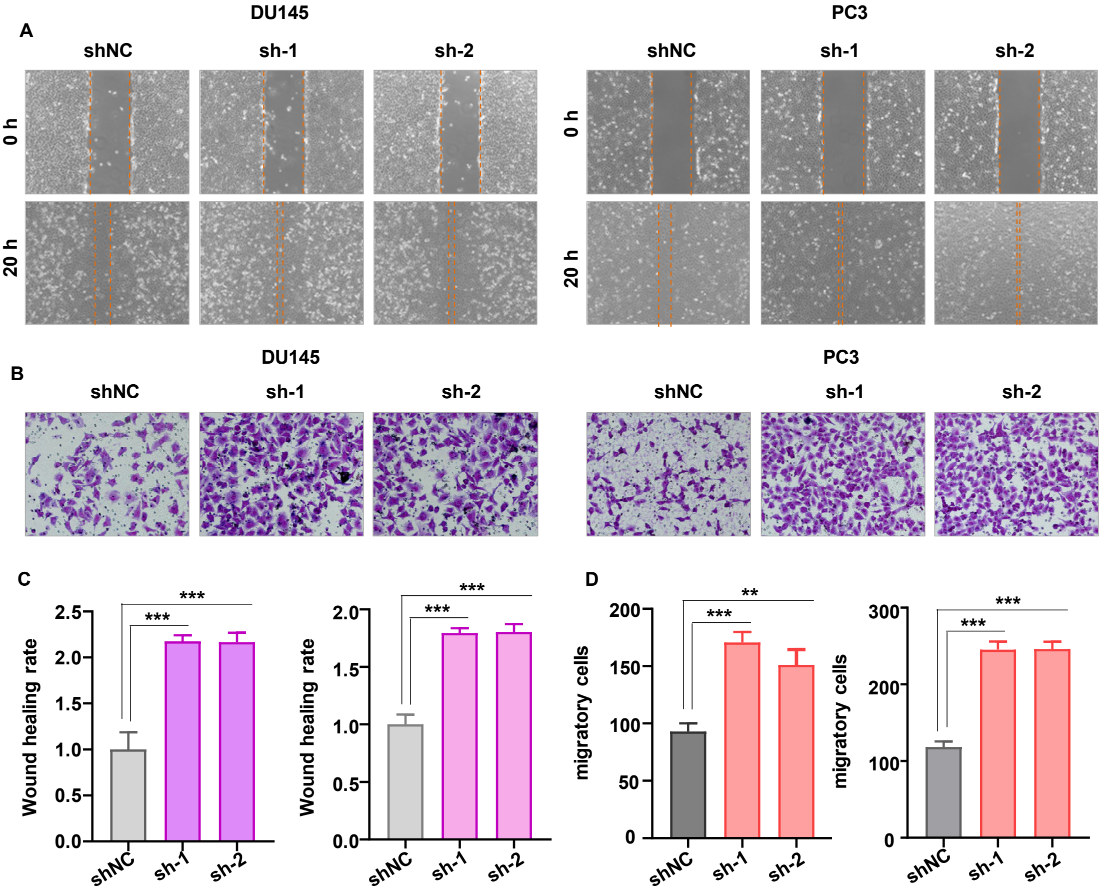

Fig. S2 Silencing of Fbxo2 facilitates cell migration in PCa.

A-D Wound-healing experiment and Transwell (without Matrigel) test were used to evaluate PC3 and DU145 cell migration capabilities after they were treated with NC shRNA or Fbxo2 shRNA lentivirus.

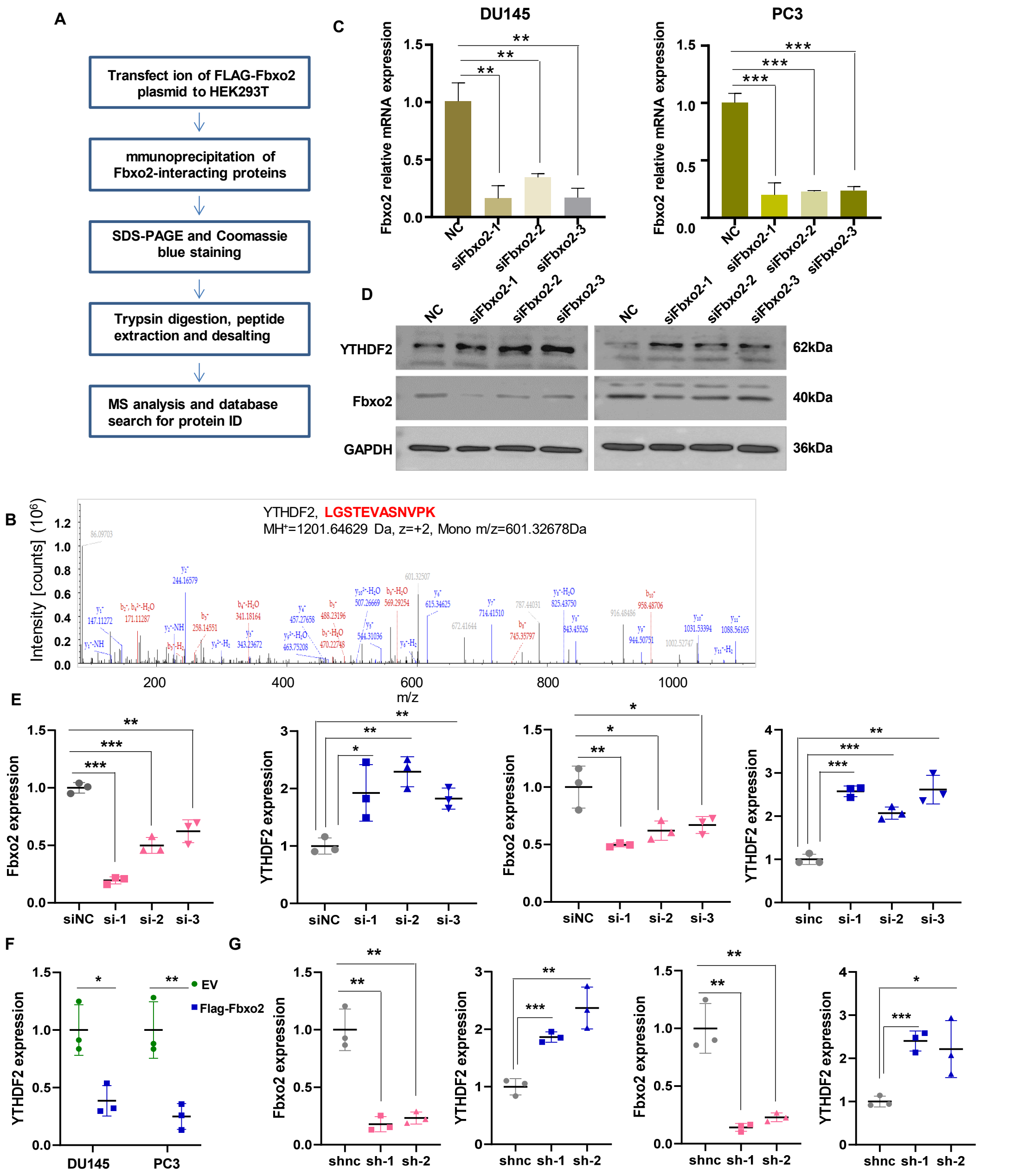

Fig. S3 Fbxo2 binds and regulates the protein stability of YTHDF2.

A Using Flag immunoprecipitates to identify Fbxo2-interacting proteins by affinity purification and LC-MS/MS method.

B A representative YTHDF2 MS/MS spectrum.

C-D The levels of Fbxo2 mRNA and protein in PC3 and DU145 cells transfected with NC siRNA or Fbxo2 siRNA are determined by RT-PCR and Western blot.

E Quantitative analysis of Fbxo2 and YTHDF2 protein levels for (**D**).

F Quantitative analysis of Fbxo2 protein levels in Figure 4F.

G Quantitative analysis of Fbxo2 and YTHDF2 protein levels in Figure 4H.

$*p < 0.05$, $**p < 0.01$, $***p < 0.001$.

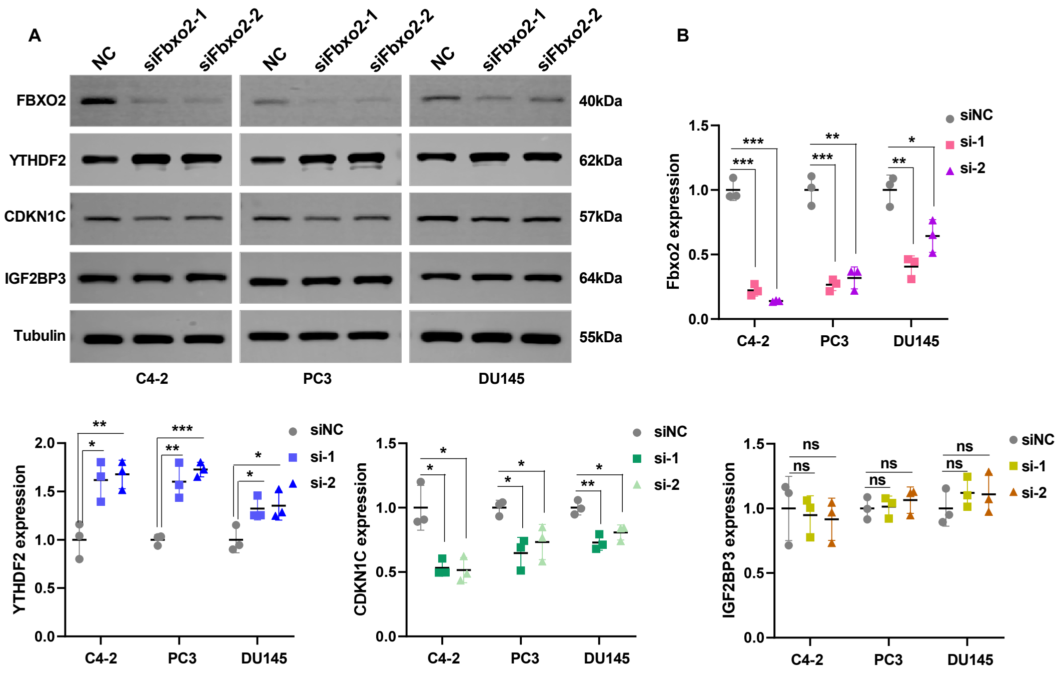

Fig. S4 Fbxo2 knockdown does not change IGF2BP3 levels.

A Knockdown Fbxo2 in prostate cancer cell lines C4-2, PC3, and DU145, and detect the protein expression of YTHDF2, CDKN1C, and IGF2BP3.

B Quantitative analysis of Fbxo2, YTHDF2, CDKN1C and IGF2BP3 protein levels for panel **A**. *$p < 0.05$, **$p < 0.01$, ***$p < 0.001$.

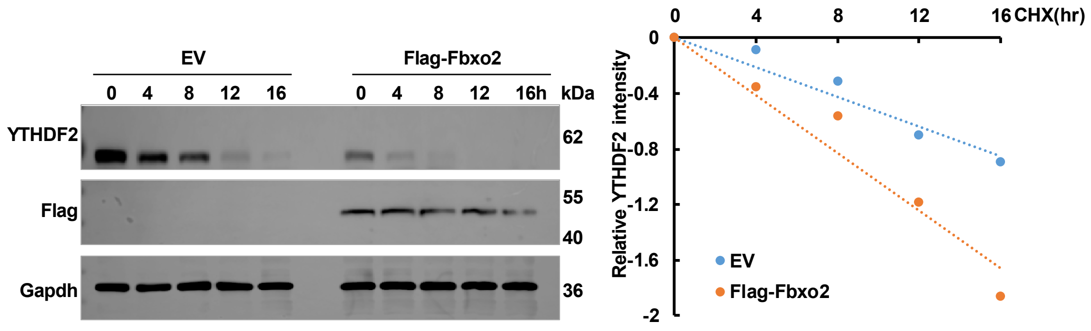

Fig. S5 Fbxo2 regulates half-life of YTHDF2 in C4-2 cells.

The protein half-life of YTHDF2 in C4-2 cells with or without Fbxo2-overexpression was identified by western blotting analysis.

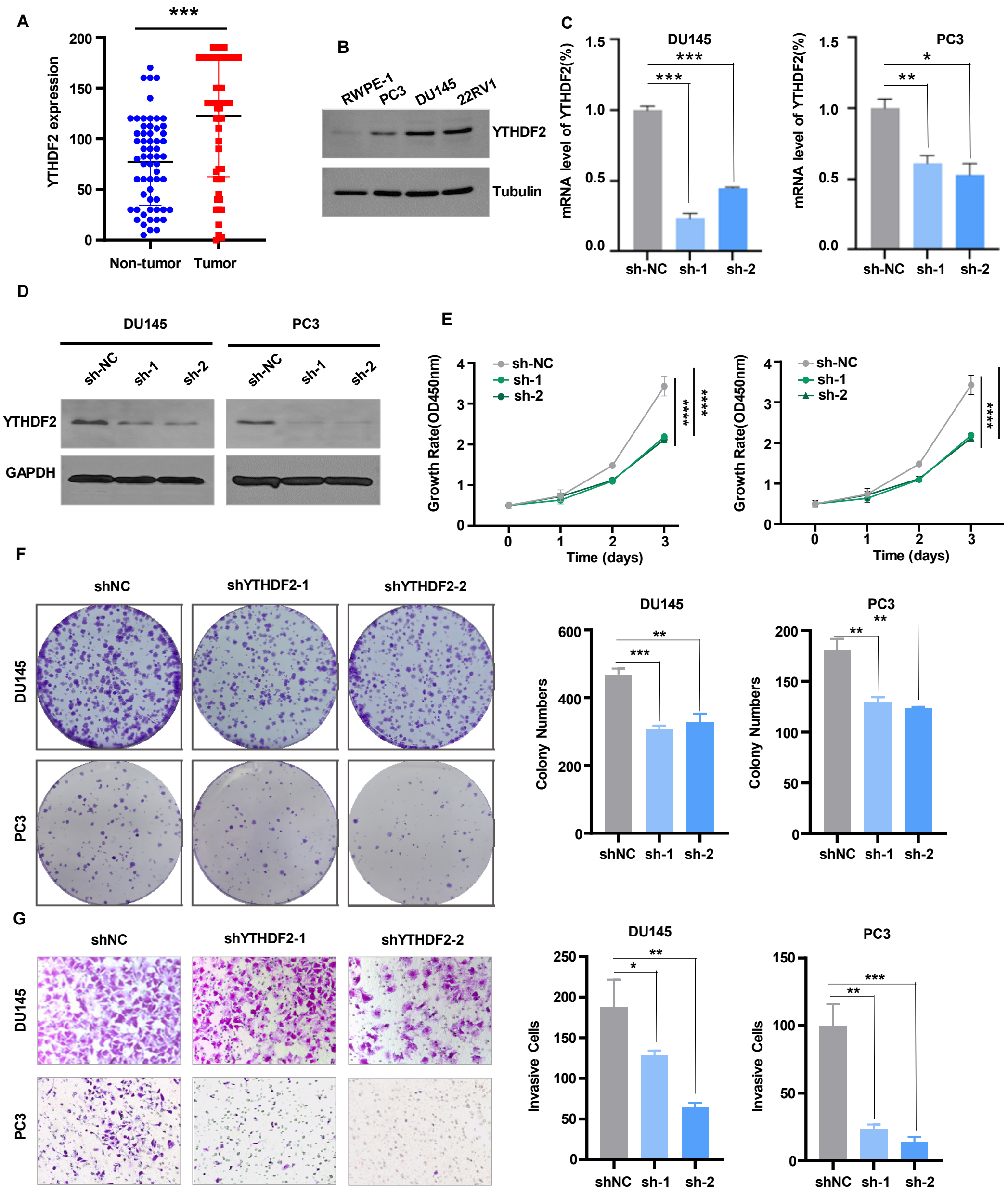

Fig. S6 YTHDF2 is highly expressed in PCa tissues and facilitates PCa cell proliferation and motility.

A YTHDF2 expression scores in 60 PCa and associated para-carcinoma samples.

B Western blotting was used to assess the expression of the YTHDF2 protein in several PCa cell lines or the human normal prostate epithelial cell line RWPE-1.

C-D RT-PCR and Western blot are used to determine the YTHDF2 mRNA and protein levels of PC3 and DU145 cells transfected with NC shRNA or YTHDF2 shRNA lentivirus.

E-F The cell viability of PC3 and DU145 cells with or without YTHDF2 silencing using CCK8 (E) and colony formation (F) tests.

G Following treatment with NC shRNA or YTHDF2 shRNA lentivirus, the Transwell assay was used to evaluate PC3 and DU145 cell capacity for invasion. $*p < 0.05$, $**p < 0.01$, $***p < 0.001$.

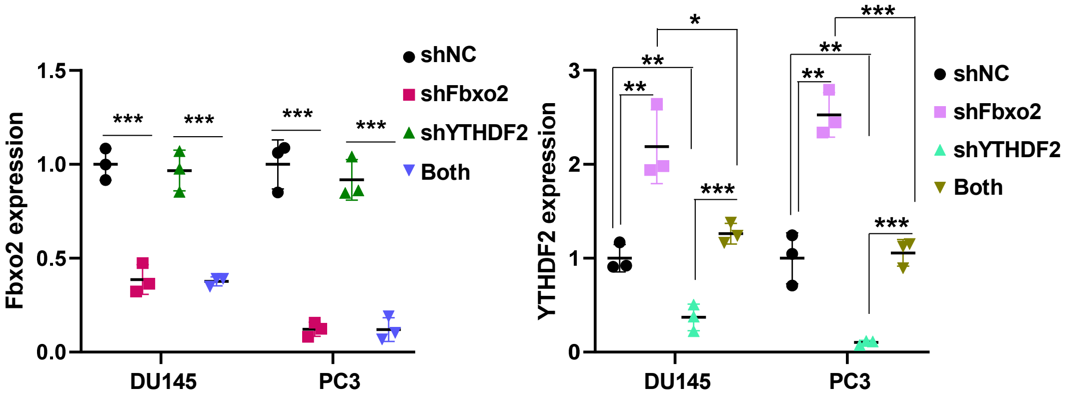

Fig. S7 Fbxo2 knockdown increases YTHDF2 expression.

A Quantitative analysis of Fbxo2 and YTHDF2 protein levels in Figure 6A. *$p < 0.05$, **$p < 0.01$, ***$p < 0.001$.

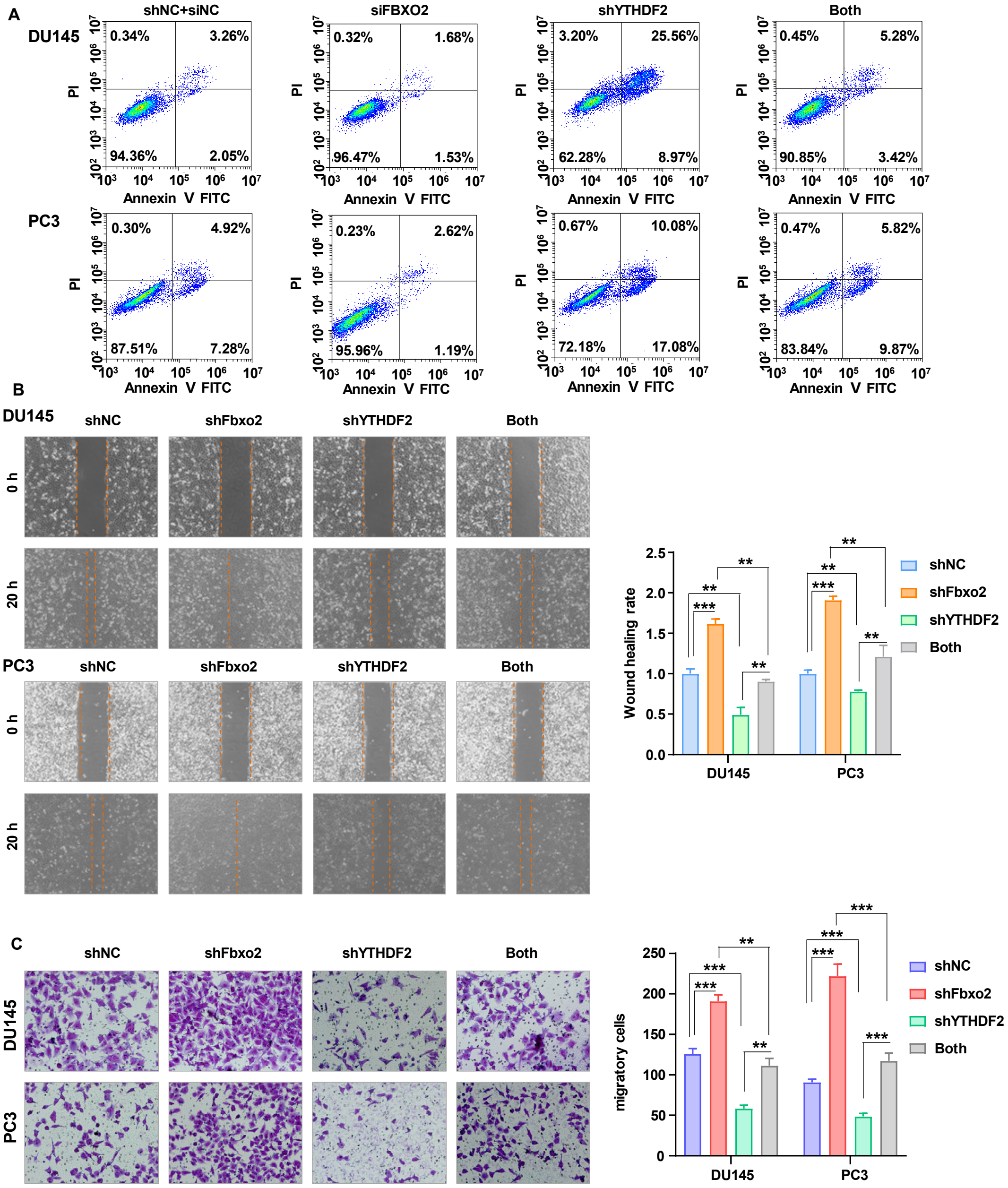

Fig. S8 YTHDF2 knockdown partially abolishes the effects of Fbxo2 knockdown on PCa cell apoptosis and migration.

A The apoptosis rate of PC3 and DU145 cells transfected with siFbxo2 alone, shYTHDF2 alone, or siFbxo2 co-transfected with shYTHDF2.

B-C Wound-healing and Transwell (without Matrigel) tests were used to evaluate PC3 and DU145 migration capabilities after they were treated with NC shRNA, Fbxo2 shRNA, YTHDF2 shRNA and Fbxo2 shRNA+YTHDF2 shRNA. *$p < 0.05$, **$p < 0.01$, ***$p < 0.001$.

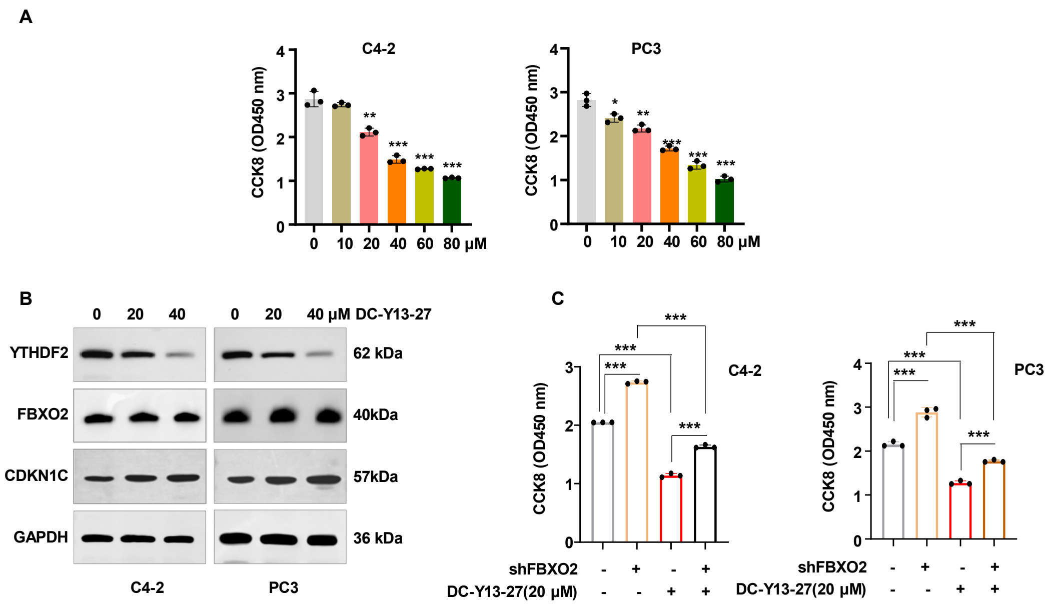

Fig S9: DC-Y13-27 inhibits the proliferation of prostate cancer cells.

A. Effect of DC-Y13-27 on PCa cells growth was detected by CCK8 assay after treatment with DC-Y13-27 for 48h.

B. Protein expression levels of YTHDF2, FBXO2 and CDKN1C in PCa cells after treatment with DC-Y13-27.

C. Assessment of cell proliferation using CCK8 assay in PCa cells after treatment with shFBXO2 and DC-Y13-27.

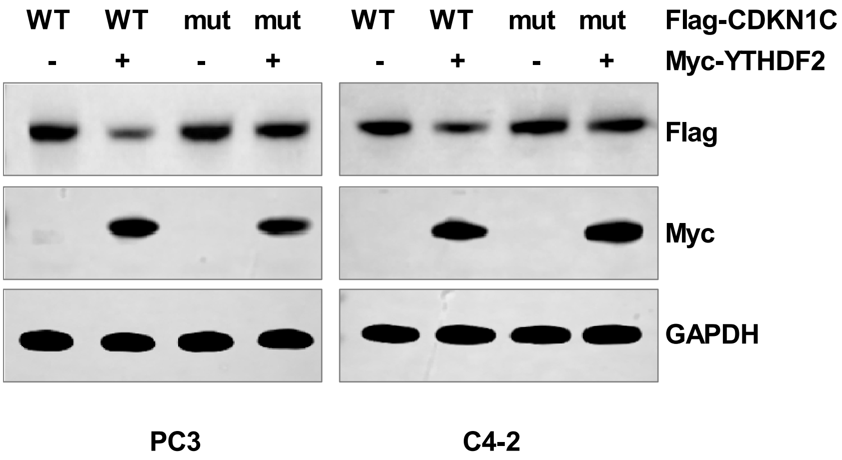

Fig S10: YTHDF2 overexpression markedly reduced the protein level of WT CDKN1C. PC3 and C4-2 cells were transfected with plasmid overexpressing CDKN1C CDS 3-UTR with either wild type (WT) or mutant (mut, A-to-T mutation) m6A sites, and the protein levels of CDKN1C were detected by Western blotting.

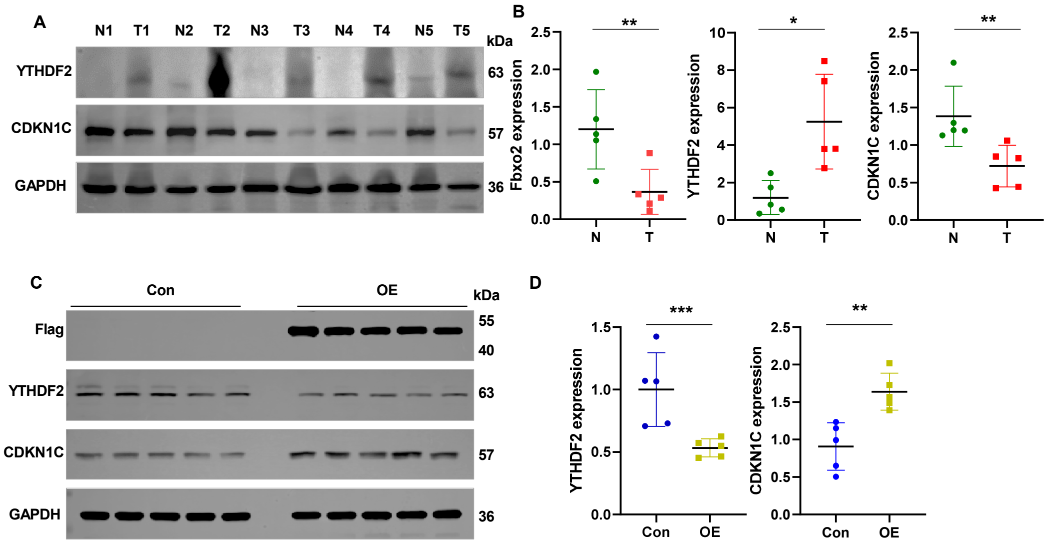

Fig S11: The expression of CDKN1C in PCa tissues and xenograft tumors.

A Immunoblot analysis of YTHDF2 and CDKN1C protein levels in freshly collected PCa tissues and matched adjacent normal tissues.

B Quantitative results for panel A.

C Western blot analysis of YTHDF2 and CDKN1C protein levels in excised xenograft tumors.

D Quantitative results for panel C.

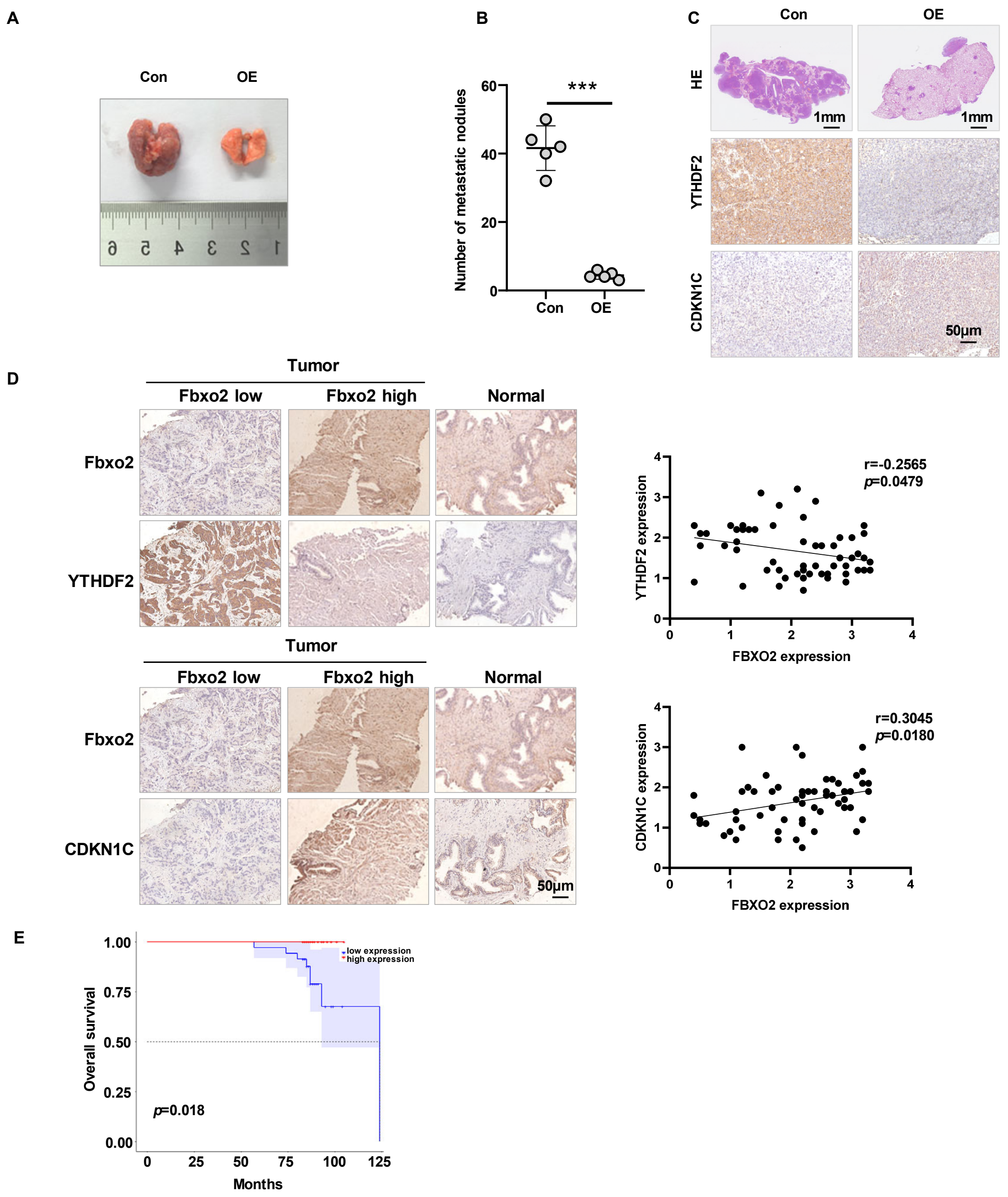

Fig. S12: Fbxo2 expression is associated with prognosis of PCa patients.

A Photographs of dissected organs.

B Quantitative analysis of lung metastasis nodules from corresponding mice.

C The levels of YTHDF2 and CDKN1C were measured in these metastatic nodules using immunohistochemistry.

D IHC staining of human the prostate cancer tissue arrays shows that YTHDF2 expression is negatively correlated with Fbxo2 expression, and CDKN1C expression is positively correlated with Fbxo2 expression.

E The correlation between Fbxo2 and the prognosis of prostate cancer patients.
