# Supplementary material for: Fbxo2 suppresses prostate cancer progression by regulating YTHDF2 ubiquitination and degradation
Source: Cell Death Dis. 2025 Dec 29;17(1):153. doi: 10.1038/s41419-025-08396-0 (PMC12858993; doi:10.1038/s41419-025-08396-0)
Supplement: Supplementary file 2 — Supplementary tables [file 41419_2025_8396_MOESM2_ESM.docx]

**Supplementary tables**

**Table S1.** Sequences of shRNAs.

| **shRNA** | **Sequence (5'-3')** |
| --- | --- |
| Scramble | CCGGCAACAAGAT GAAGAGCACAACTCGAGTTGGTGCTCTTCATCTTGTTGTTTTT |
| shFBXO2-1 | CACCGTTAAGCTACTGTCCGAGCACGAGAACGTGCTGGCTGAGTTCAGCATTTTTT |
| shFBXO2-2 | TCGTGGTGAAGGACTGGTACTCGGGCCGCAGCGACGCTGGTTGCCTCTACTTTTT |
| shYTHDF2-1 | AGTTGGCTATTGGGAACGTCCTTCAAGAGAGGACGTTCCCAATAGCCAACTTTTTTT |
| shYTHDF2-2 | GCACAGAAGTTGCAAGCAATGTTCAAGAGACATTGCTTGCAACTTCTGTGCTTTTTT |

**Table S2.** Sequences of siRNAs.

| **siRNA** | **Sequence (5'-3')** |
| --- | --- |
| siRNA_NC | F: UUCUCCGAACGUGUCACGUTT |
|  | R: ACGUGACACGUUCGGAGAATT |
| siFBXO2-1 | F: GCCGCAACCUUCUGCGUAATT |
|  | R: UUACGCAGAAGGUUGCGGCTT |
| siFBXO2-2 | F: GUGACCAACAGCAGCGUGUTT |
|  | R: ACACGCUGCUGUUGGUCACTT |
| siFBXO2-3 | F: GCGACGCUGGUUGCCUCUATT |
|  | R: UAGAGGCAACCAGCGUCGCTT |

**Table S3.** Primer sequences for RT-qPCR and ChIP-qPCR.

| **Genes** | **Sequence (5'-3')** |
| --- | --- |
| *FBXO2* | F: GATGAGAGCGTCAAGAAGTACT |
|  | R: GGACAGTAGCTTAACGGTGAG |
| *GAPDH* | F: GGAGCGAGATCCCTCCAAAAT |
|  | R: GGCTGTTGTCATACTTCTCATGG |
| *YTHDF2* | F: GTGAGAGCCTGTCGAGCATCACT |
|  | R: ACAGGCATGCAACACCATGCAG |
| *CDKN1C* | F: CTGATCTCCGATTTCTTCGC |
|  | R: TCTTTGGGCTCTAAATTGG |
| *CDKN1C* F1  （P.735=CDKN1C） | F: CCGACGCAGAAGAGTCCAC |
|  | R: CGAGAAGAAGGGGAAAGGAGAG |
| *CDKN1C* F2  （P.735=CDKN1C） | F: GGCCAAGTGCGCTGTGCTC |
|  | R: TCCCACGGGCGACAAGACG |
| *CDKN1C* F3  （P.999-P.1134=CDKN1C） | F: CTTCTCGCTGTCCTCTCCTCTCTC |
|  | R: GAGGCCGTCGAGGGACTCAG |
| *CDKN1C* F4  （P.2534=CDKN1C） | F: TCGCAGTTTAGAGCCCAAAGAG |
|  | R: AACAAAACCGAACGCTGCTC |

**Table S4. clinical information for 60 PCa patients**.

| Age | Cases |  |
| --- | --- | --- |
| ≤67.5 | 28 |  |
| >67.5 | 32 |  |
| Gleason |  |  |
| ≤6 | 13 |  |
| ＞6 | 47 |  |
| Prognosis |  |  |
| No Recur | 14 |  |
| Recur | 46 |  |
