# Supplementary material for: Fbxo2 suppresses prostate cancer progression by regulating YTHDF2 ubiquitination and degradation
Source: Cell Death Dis. 2025 Dec 29;17(1):153. doi: 10.1038/s41419-025-08396-0 (PMC12858993; doi:10.1038/s41419-025-08396-0)

Fig 1G

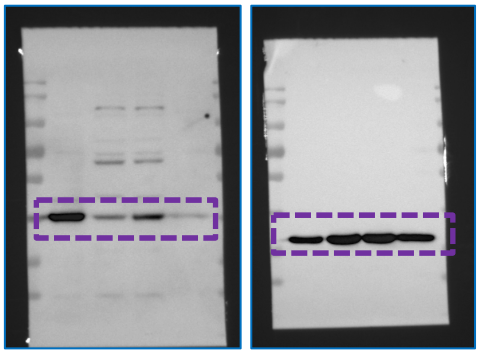

Fig 1H

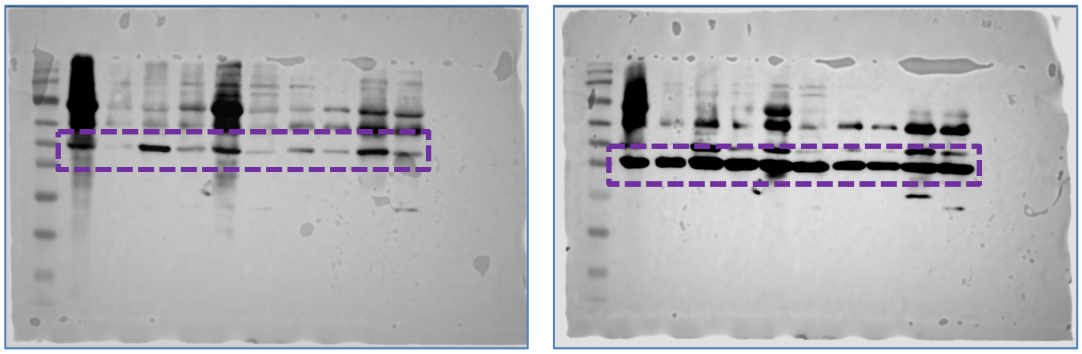

Fig 2A

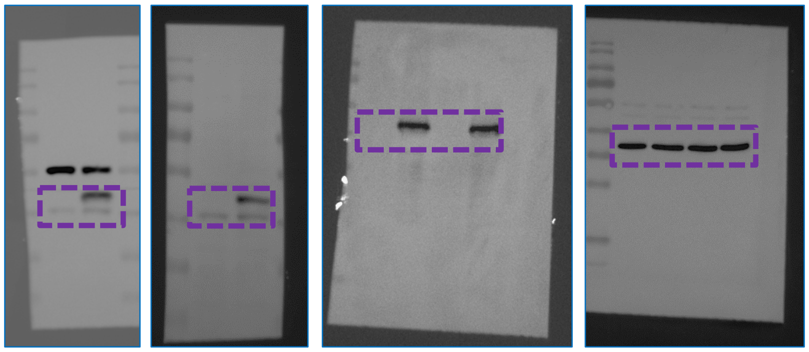

Fig 2L

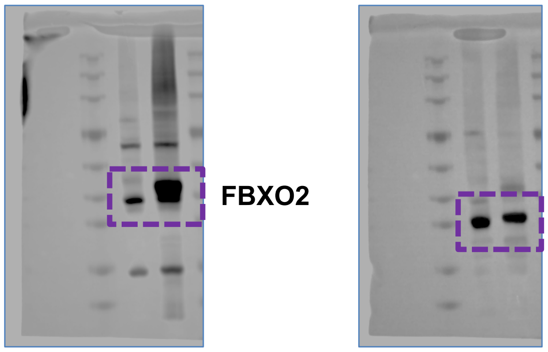

Fig 2I

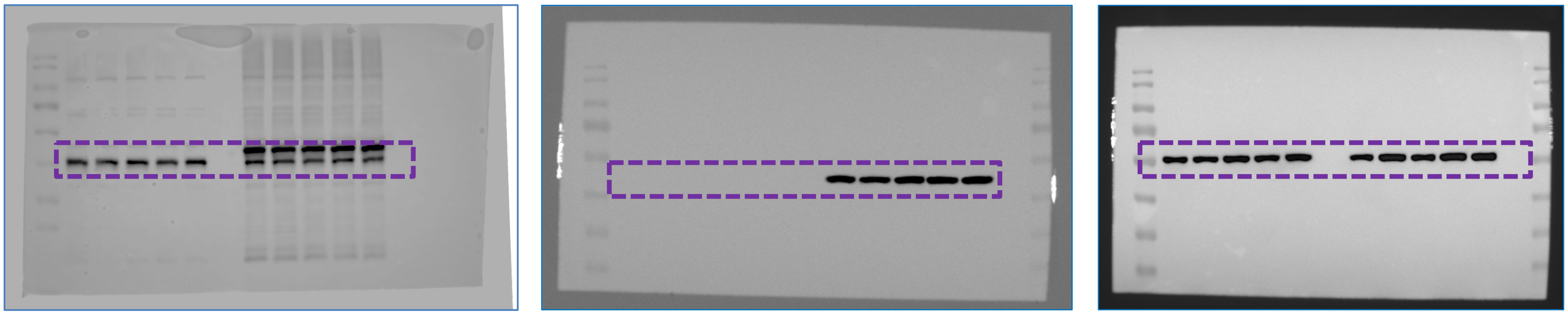

Fig 4B

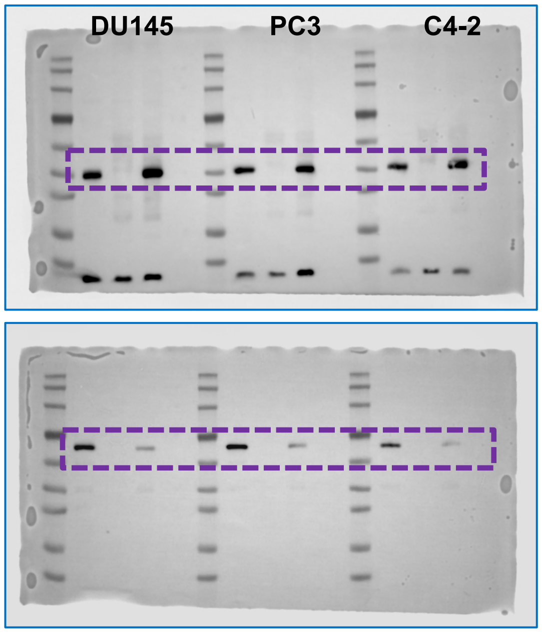

Fig 3A

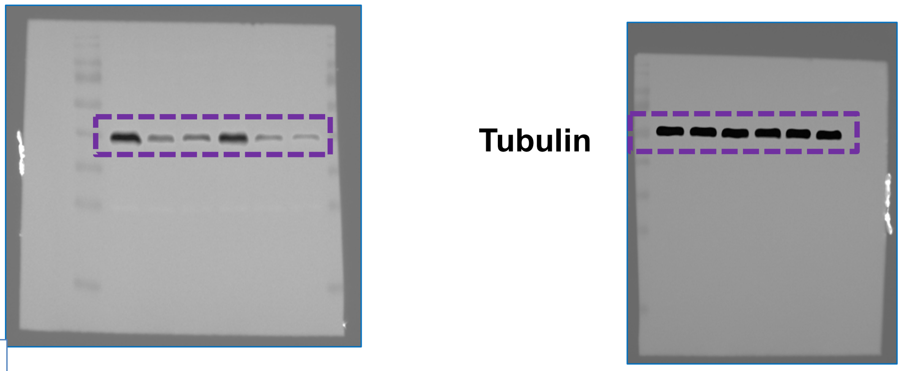

Fig 4C

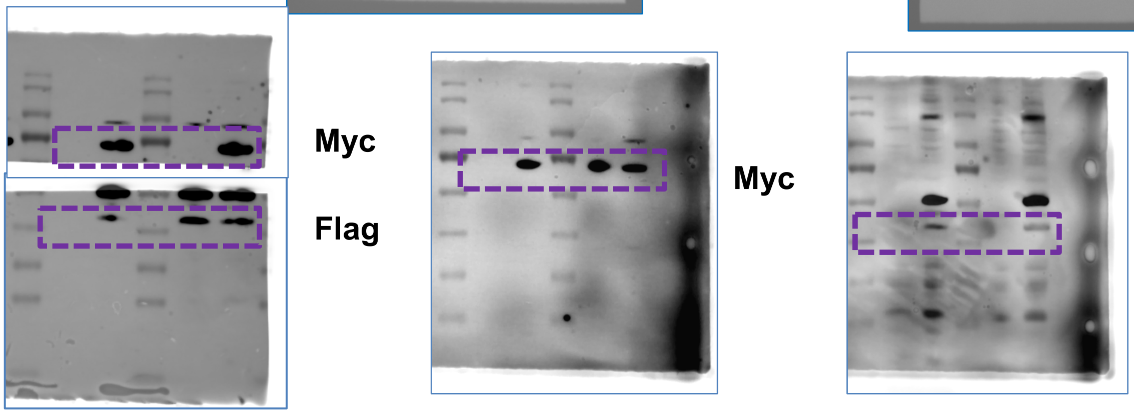

Fig 4E

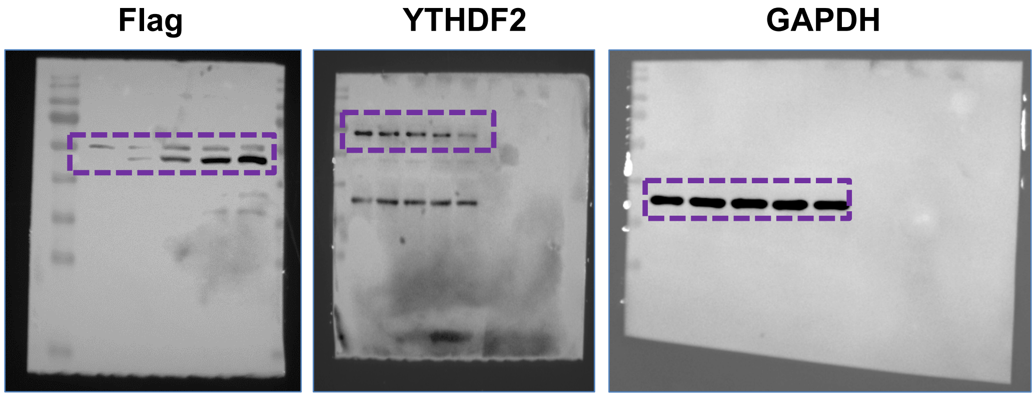

Fig 4F

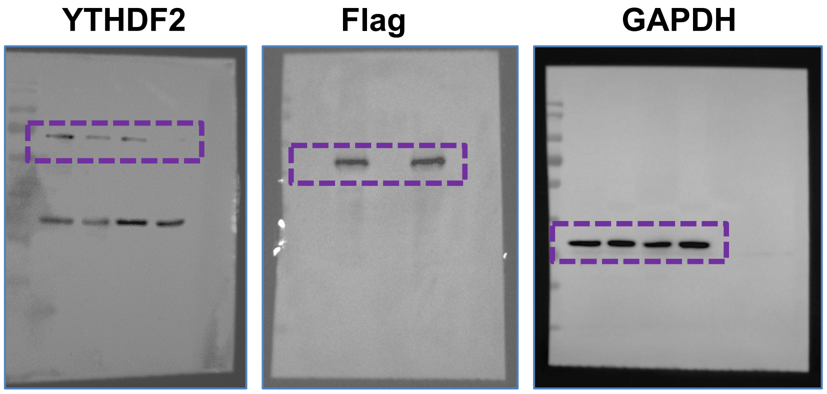

Fig 4H

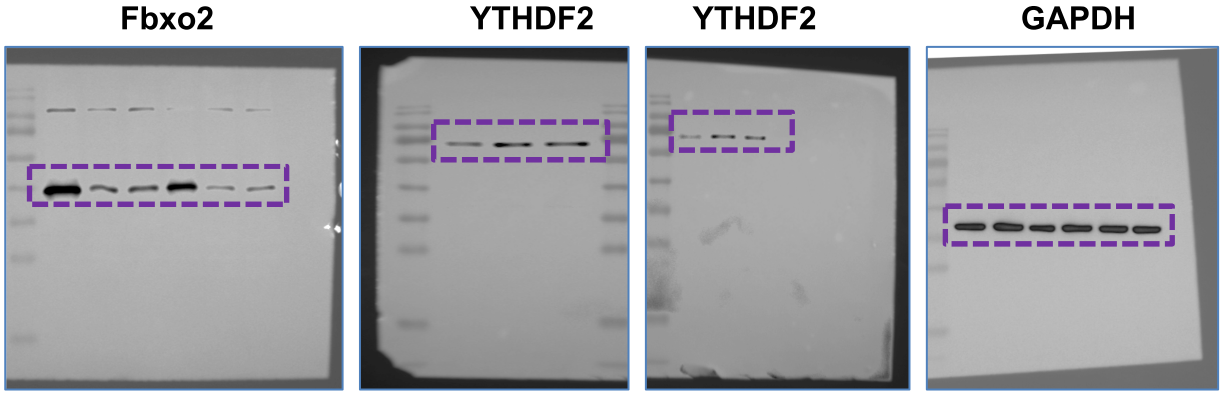

Fig 5A

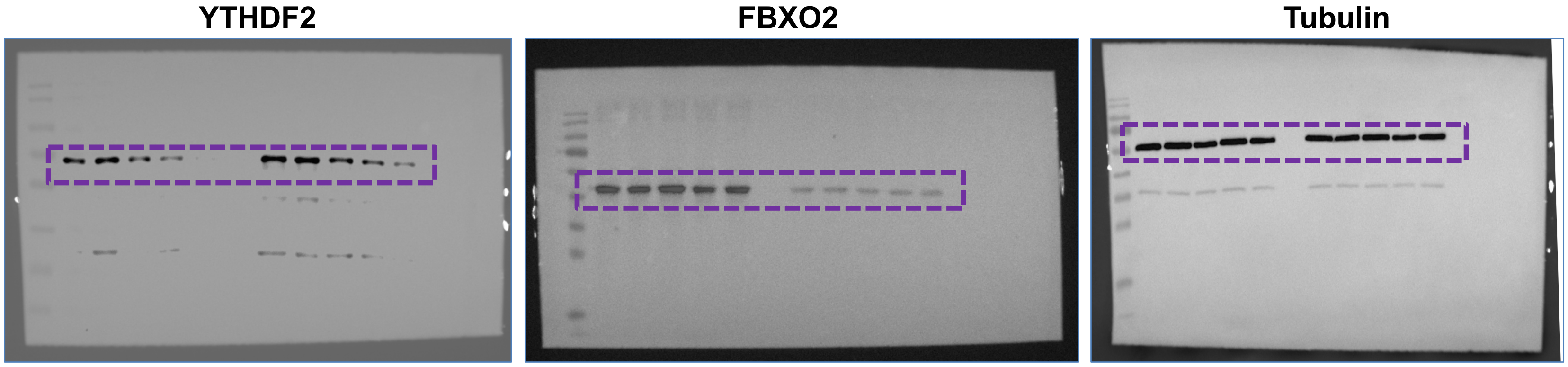

Fig 5C

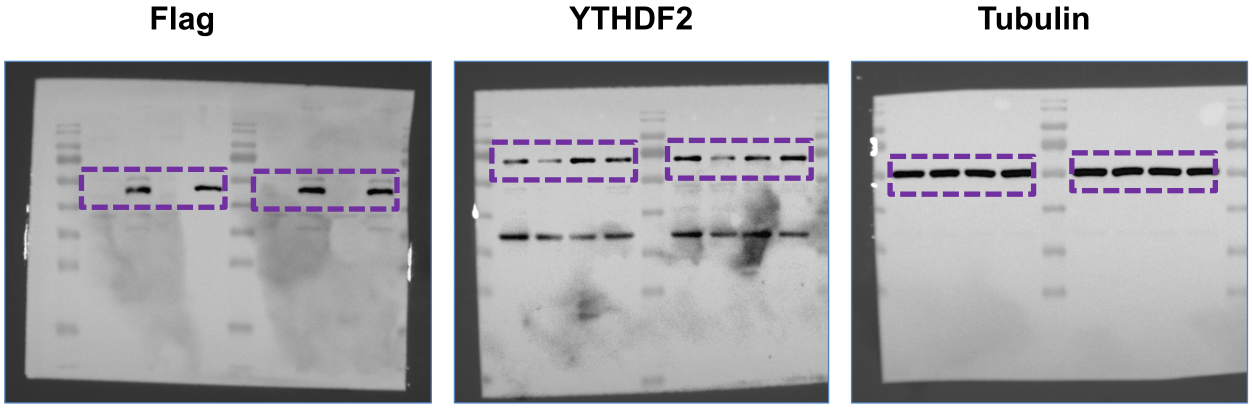

Fig 5D

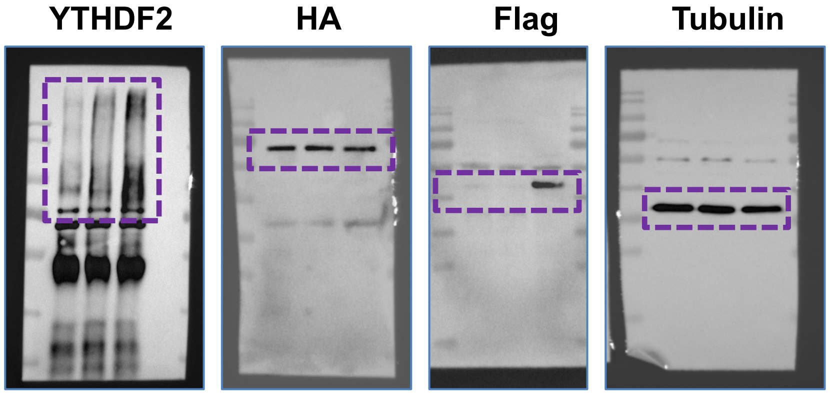

Fig 5F

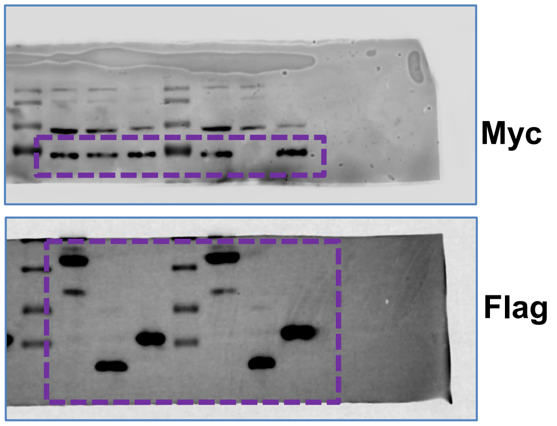

Fig 7I

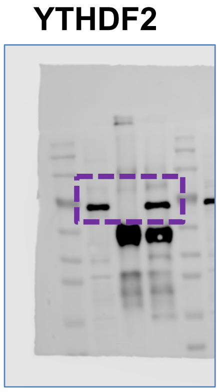

Fig 5H

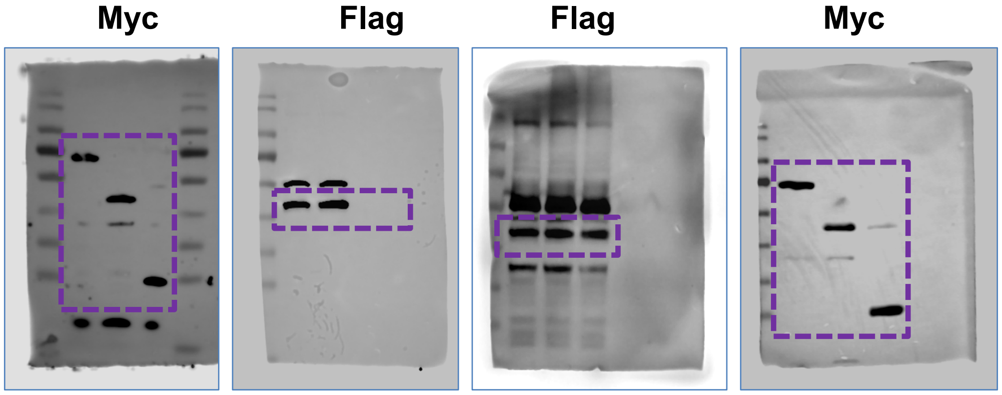

Fig 5G

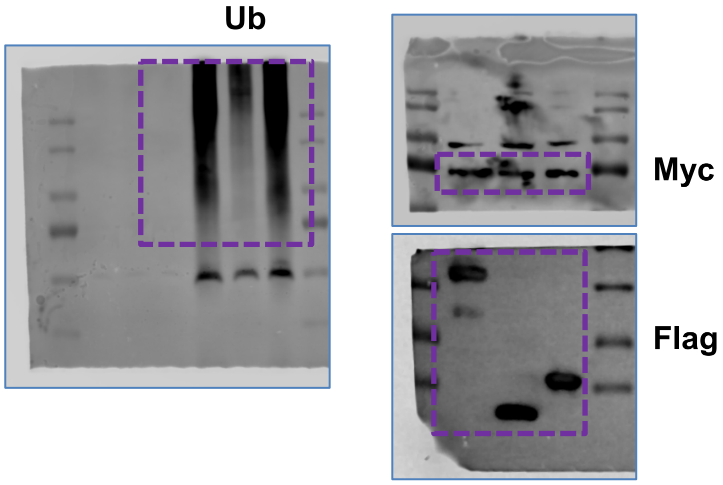

Fig 5I

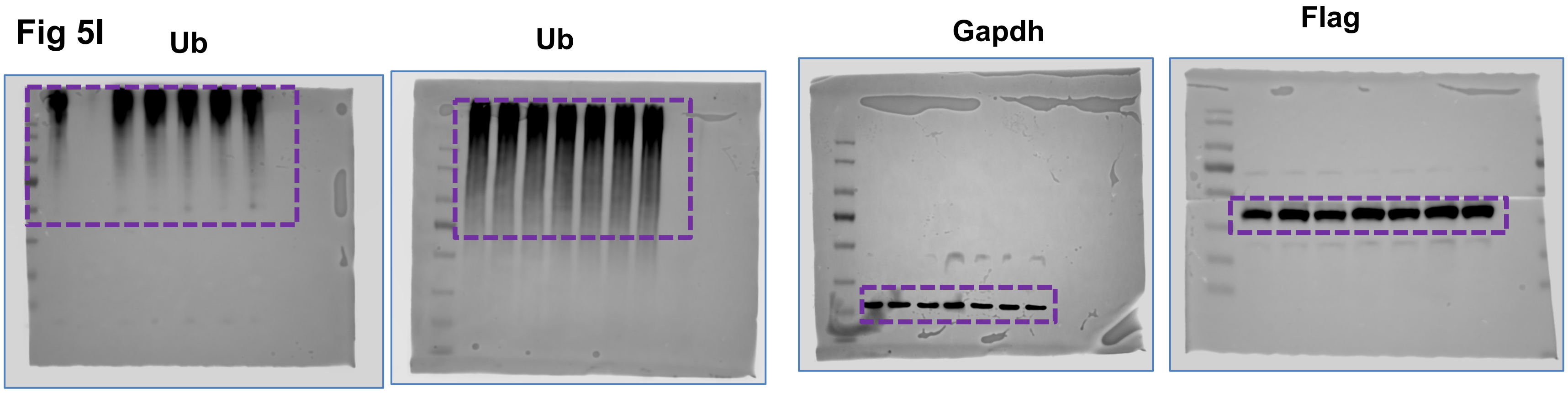

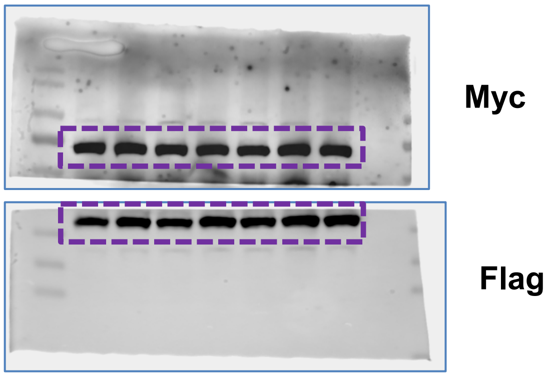

Fig 6A

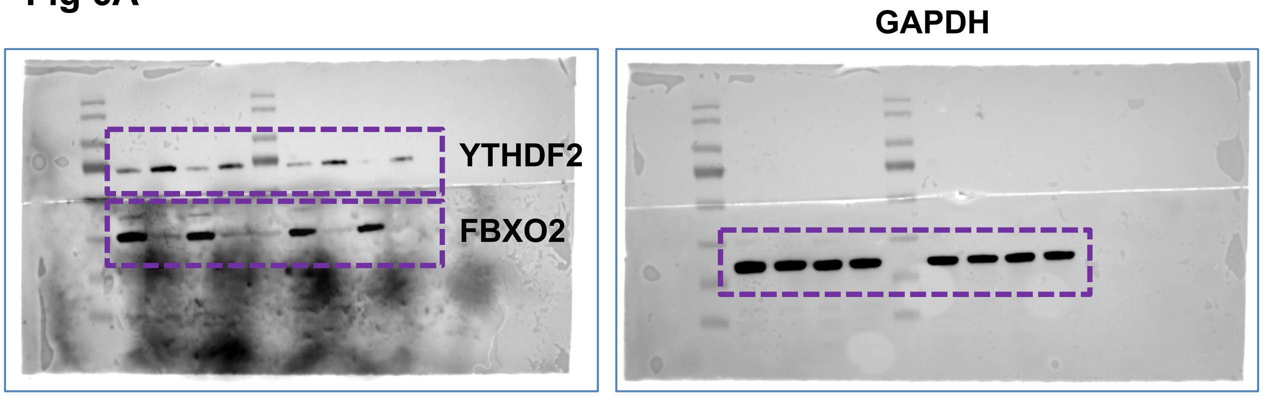

Fig S3D

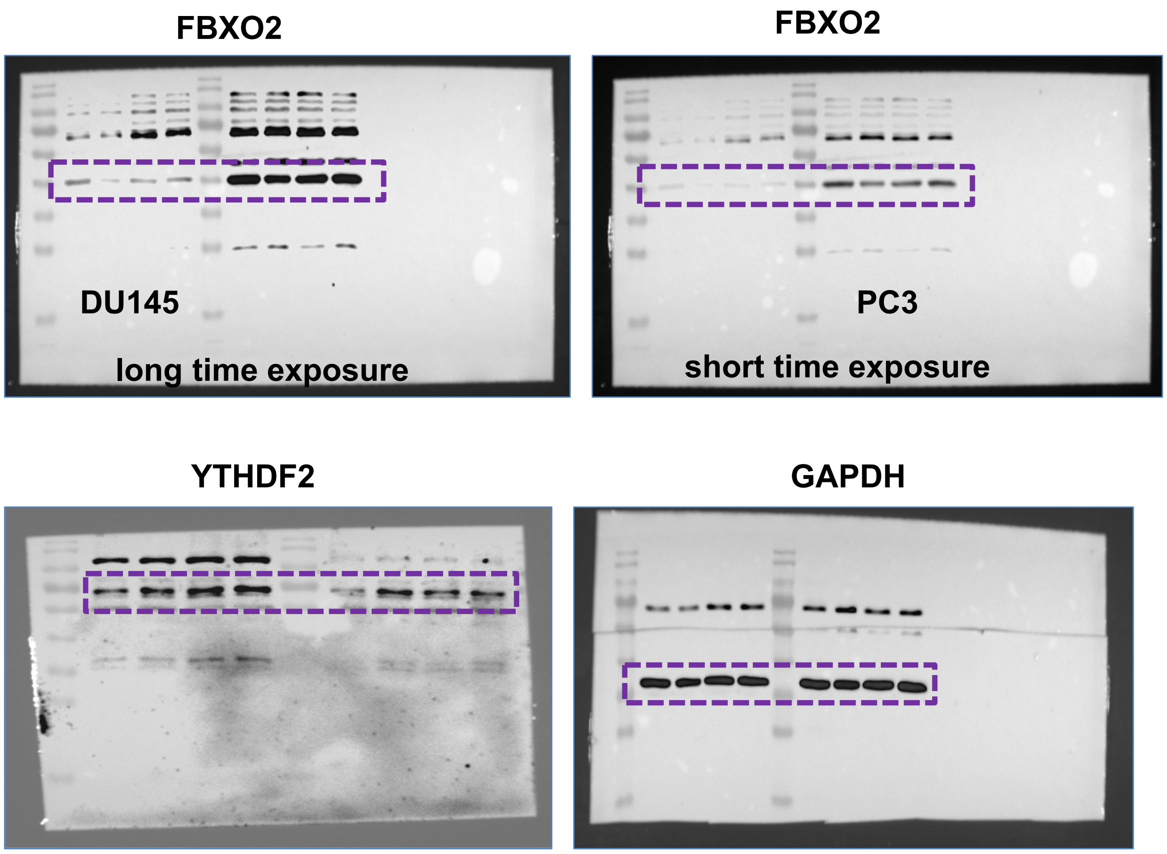

Fig S4A

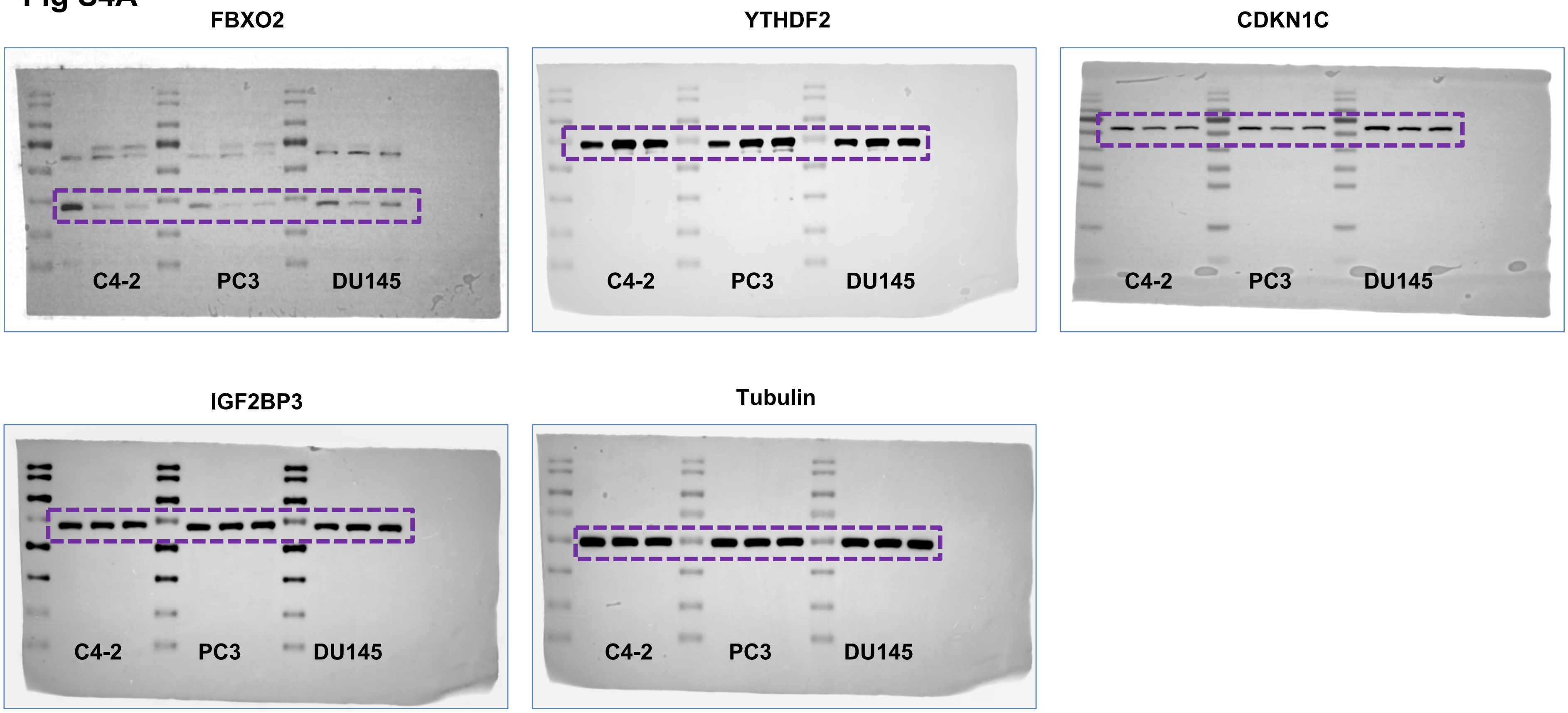

Fig S5

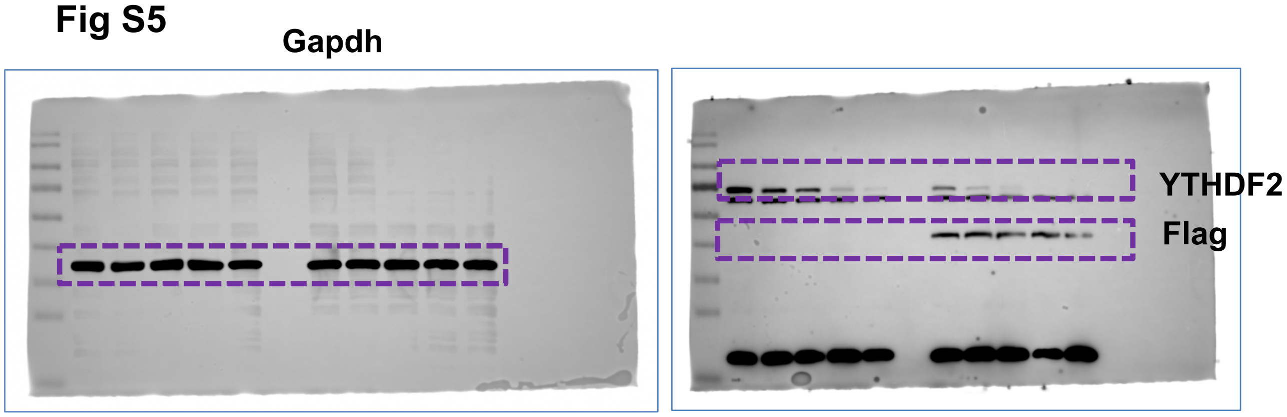

Fig S6B

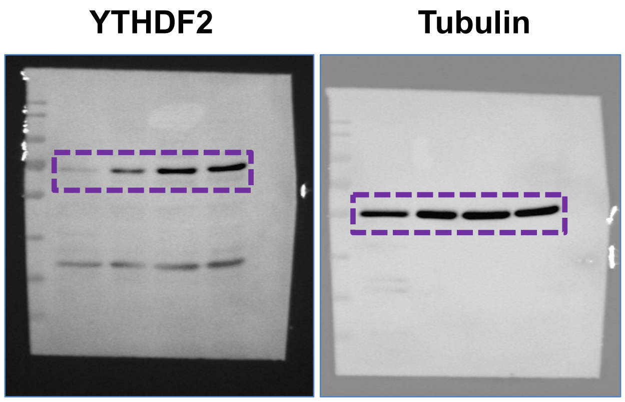

Fig S6D

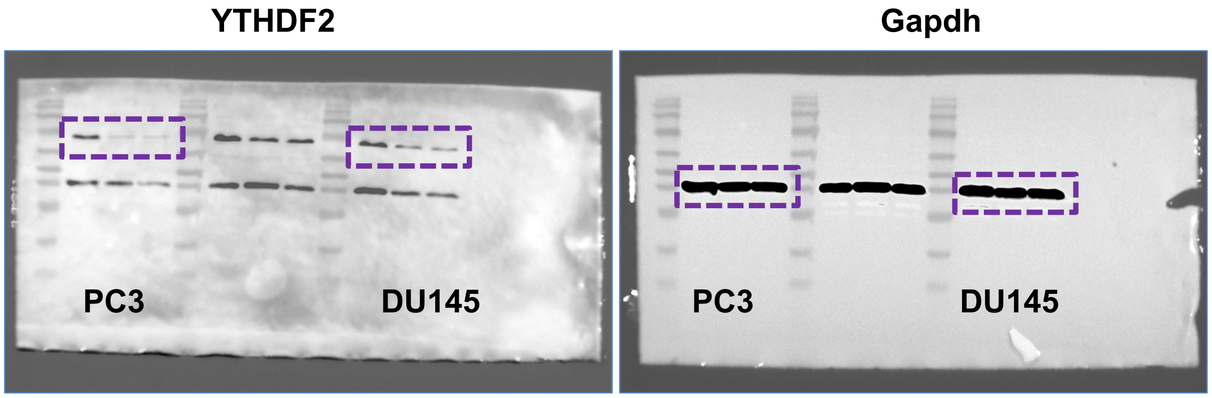

Fig S9B

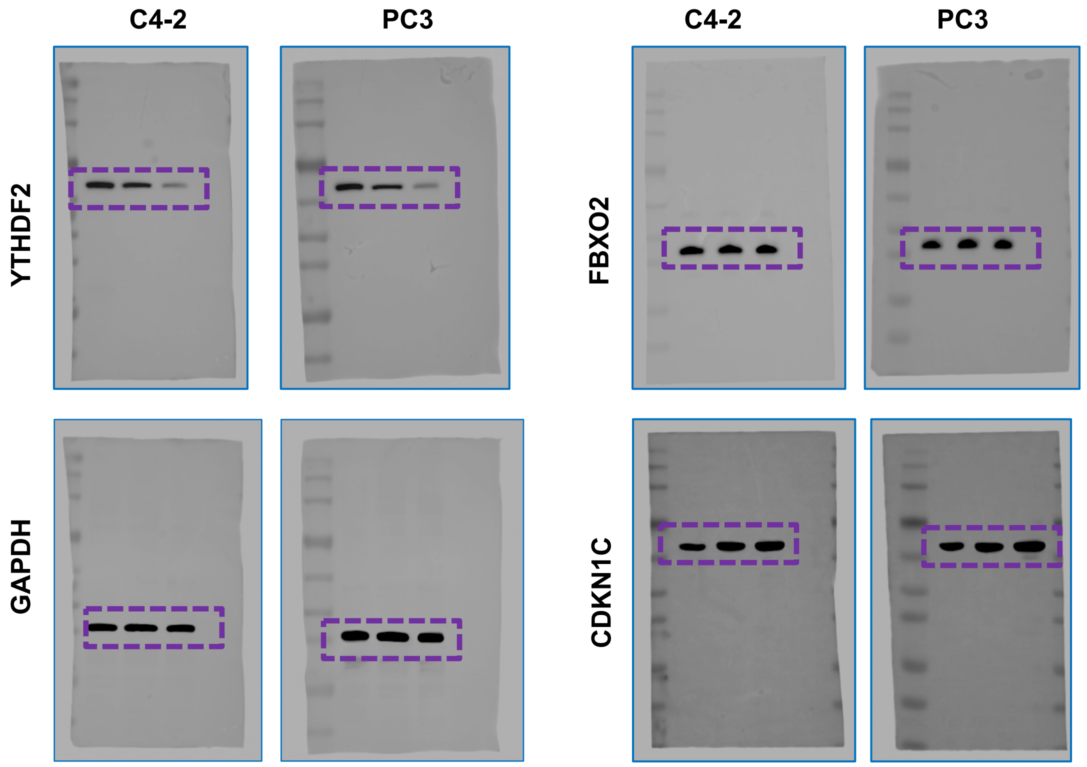

Fig S10

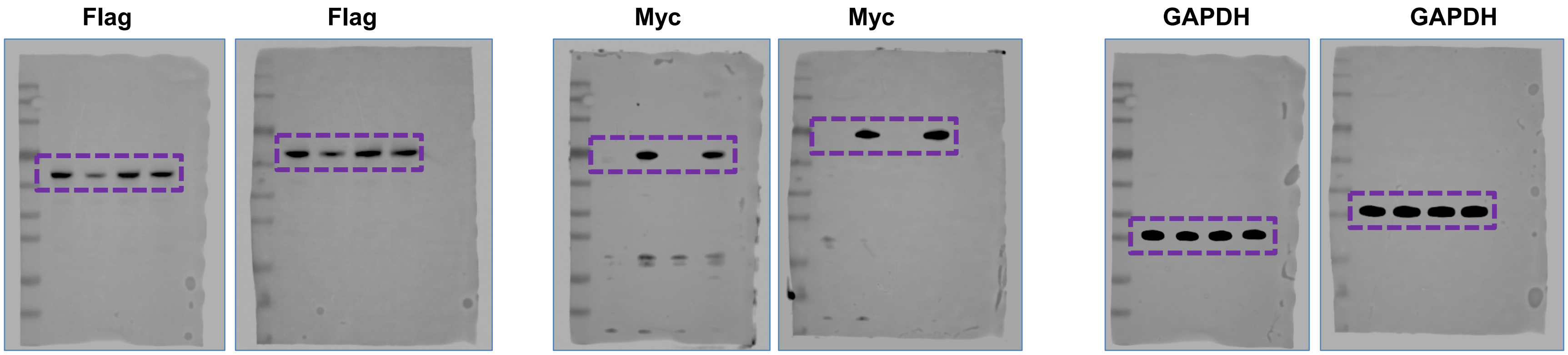

Fig S11A

YTHDF2

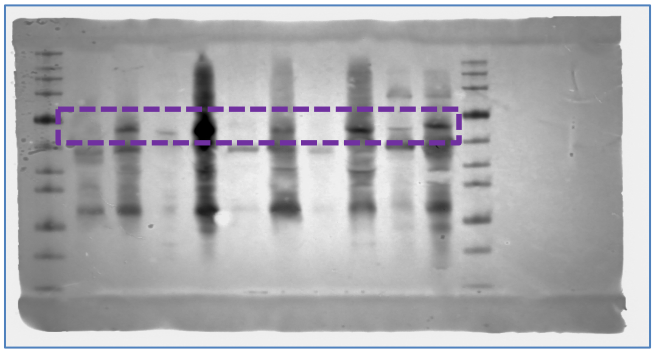

GAPDH

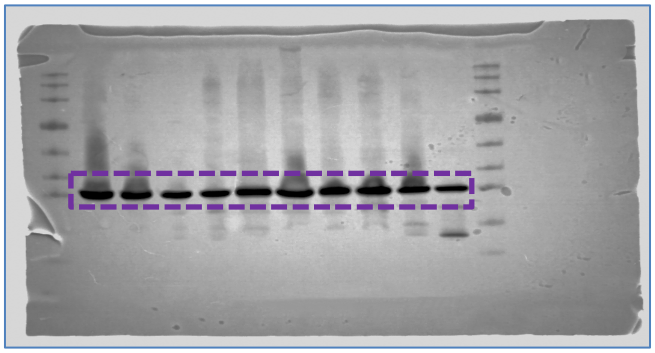

CDKN1C

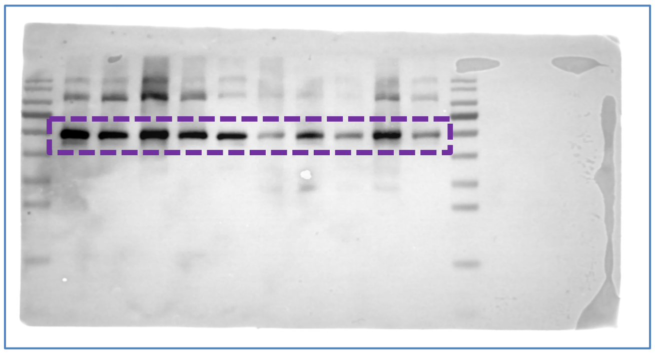

Fig S11C

Flag

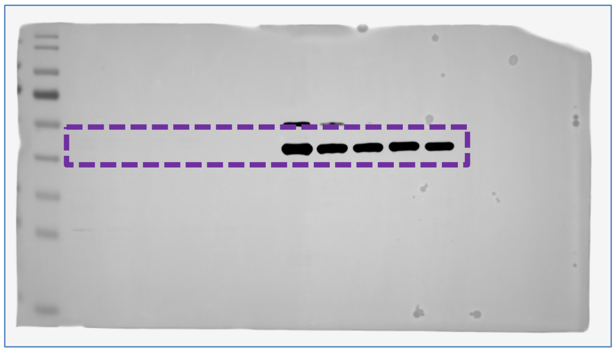

YTHDF2

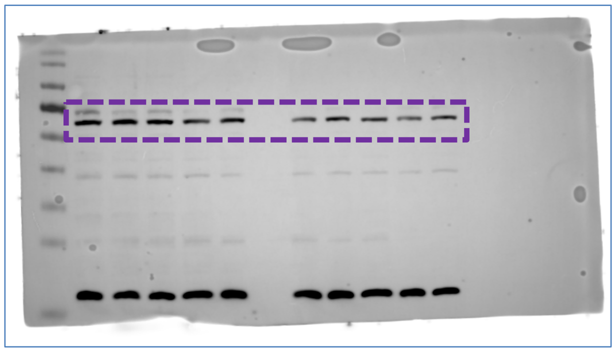

CDKN1C

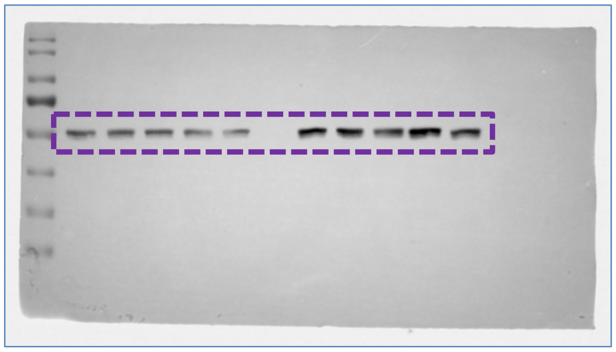

GAPDH

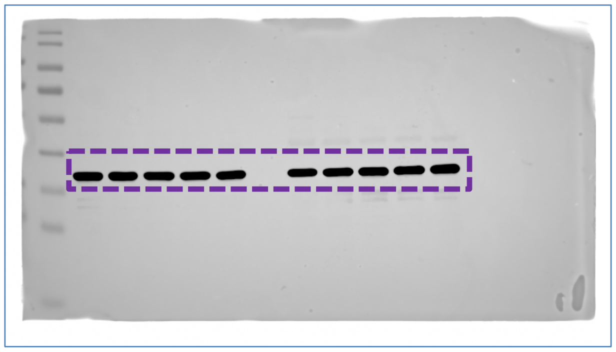

Supplement: Supplementary file 3 — Original Western blotting images [file 41419_2025_8396_MOESM3_ESM.pdf]
